# Smart transactive energy based approach for planning and scheduling in multi-looped microgrid distribution network across planning horizon

**DOI:** 10.1016/j.heliyon.2024.e25408

**Published:** 2024-02-16

**Authors:** Mustafa Tariq, Syed Ali Abbas Kazmi, Abdullah Altamimi, Zafar A. Khan, Bader Alharbi, Hamoud Alafnan, Halemah Alshehry

**Affiliations:** a US-Pakistan Center for Advanced Studies in Energy (USPCAS-E), National University of Sciences and Technology (NUST), H-12, Islamabad, 44000, Pakistan; b Department of Electrical Engineering, College of Engineering, Majmaah University, Al-Majmaah, 11952, Saudi Arabia; c Engineering and Applied Science Research Center, Majmaah University, Al-Majmaah, 11952, Riyadh, Saudi Arabia; d Department of Electrical Engineering, Mirpur University of Science and Technology, Mirpur, AJK, 10250, Pakistan; e Department of Electrical Engineering, College of Engineering, University of Ha'il, Ha'il, 55476, Saudi Arabia; f Department of Educational Sciences and Design, College of Education, Majmaah University, Al-Majmaah, 11952, Saudi Arabia

**Keywords:** Distribution network, Electric systems, Micro-grid, Power system planning, Power system scheduling, Renewables, Transactive energy

## Abstract

In this paper, an innovative transactive energy approach is proposed as viable option for coordinated distribution system planning across a certain horizon. The proposed approach is evaluated across a multi-looped (meshed) test system and is implemented with load growth having prosumers participating in the electrical market in transactive energy system aiming at evaluation on techno-economic basis. Apart from prosumer sensitivity analysis, evaluations have been carried across reducing total production cost of energy, reduction in per unit price, active power losses. Whereas improving voltage profile, cost of scheduling and consumer per kWh purchase and sales in comparison with traditional counterpart. The proposed framework includes optimization algorithm aiming at sources scheduling and IEEE 69 system for validation. The algorithm minimizes cost, maximizes energy efficiency, increases renewable energy mix and reduces consumers cost of energy purchase. Reduction of 51.44 % in cost of energy is achieved, whereas loss reduction of 12.6% is achieved. The comparison of IEEE 69-bus base case with the 10 %, 15% and 20% transactive energy applied with simulations to evaluate performance parameters that will directly benefit both prosumers and utility alike in-terms of low bills and further reduction of stress on the grid amid load growth across multiple years.

## 1 Introduction

The smart grid technology has enabled to use energy resources and load demands more efficiently, primarily on the distribution grid end. The smart technologies of flexible load and advancement in generation technologies such as solar PV and wind has led to a path for the advancement in the power system. The flexible loads can offer controllability that can be utilized to operate in uncertainty. Moreover, Demand side management (DSM) with its variant of demand response (DR) has led to an incentivized load management such as in time-of-use (ToU) pricing. The electricity unit price pays a huge role in shifting the load, thus maintain a balance of power system more reliably and efficiently, during the high consumption or critical time. Small-scale investment of consumer for fully independent and clean energy on distribution side has open a path for the consumption side to participate in energy demands. In this end user, which has surplus amount of energy during low consumption time can participate in balancing the energy demands. In this context, a revolutionized market system “Transactive energy system” has been introduced that can be defined as “a set of economic and control mechanisms that allow the dynamic balance of supply and demand across the entire electrical infrastructure using value as a key operation parameter” [1].

The transition of electricity market i.e. transactive energy market system is introduced as a demand Response (DR) has evolved in the past few years. It can be said that transactive energy (TE) is the advancement in demand side management (DSM). In DR, only the load of the consumers is controlled as needed. Demand Response is divided into various types. 1) Direct Load control in which load controlling entities pay incentive to the customer that are willing to participate to control some of their load directly at some times. 2) Price base demand response provides time varying signals for pricing to induce customer to shift their load at low peak hours. Time varying pricing includes time-of-use tariffs (TOU), critical peak pricing (CPP), and critical peak rebate tariffs (CPR) and real time pricing tariffs (RTP).

In contrast to the conventional hierarchical grid topology, TE can be considered as an improved variant of DR that supports a network environment for decentralized nodes. The network architecture enables interaction such that all levels of energy generation and consumption are considered and can communicate with one another. From the viewpoint of TE, the ability of the associated systems to exchange information about energy while maintaining operation and limitations on services refers to as interoperability. The TE strategy has various benefits compared to the conventional energy grid, prominently greater use of grid resources, further customer satisfaction and lower energy costs. The core goal of TE remains that energy must be efficiently allocated from sellers to buyers through some sort of evaluation mechanism [2].

TE mainly focuses on more independency from the main grid. Energy requirement is meet through local microgrids (MG) and energy imbalance is catering through the combination of Microgrids and consumer load management. Various researches have been done in transactive energy in the past few years. The comprehensive energy management system (CMES) is presented that deals with energy mismatch by using techniques and distributed energy storage system (DESS) [3]. Two level optimal scheduling technique has been discussed in Ref. [4] that implement transactive energy system as virtual power plant for regulating the distributed energy resources.

In wholesale market and real time market are represented that take part in optimally scheduling DERs for maximizing profit for the participant in Day ahead market and dealing with the energy imbalance in real time market [5]. In this paper, a scheduling mechanism is proposed for competitive market consisting of multiple prosumer microgrids in which every MG is assigned with producer points (PP) for production and consumer points (CP) for consumption along with contribution metric (CM) as contribution metric. Higher the CM, higher the benefit at the cost of maximizing contribution and minimizing utilization. In Ref. [6], The local energy markets are introduced in which clearing house model is used for trading with simultaneous auctions along with price formation model for trading and mathematical models including uncertainty representation, via renewable generation, load profiles and market prices and scenarios created via Monte Carlo Simulation (MCS), and two stage stochastic model.

An agent-based test system is designed in Ref. [7] for the transactive approach to ensure reliable operations of integrated transmission and distribution (ITD) systems with growing distributed energy resources (DERs) penetration. It introduces distribution system operator (DSO) participating in ITD system, to use TE system to manage power usage of DERs in accordance with the local goals and constraint of DERS owners and to benefit from DERs in taking ancillary services by a market-based compensation process. Conditional value at risk (CVaR) to measure profit variation and Enhanced Particle Swarm Optimization algorithm (EPSO) and commercial solver to solve scheduling problem are presented in which a two-stage scheduling model for DER in Transactive Energy framework is proposed in which virtual power plant (VPP) participate in DA market at first stage to maximize profit and RT market in second stage to minimize loss. However integrated scheduling and planning framework needs to be addressed from the perspective of TE.

Andrea et al. [8] aims at the potential of home energy monitors for TE supply arrangements from the perspective of current metering arrangements and residential energy monitoring. It also describes the study of home energy monitoring in the form of high-tech supporter to unlock the local trading possibilities of the investment in micro energy generation. It led to the result by getting knowledge of consumer behavior, and by implementing effective human factors techniques that the present and future energy consumers can have an approach to the products that will be quite enough to meet the increasing requirements of energy sector. However, the trend across various variations of load, tariffs and impact on system losses needs further consideration.

As per Ron [9], TE system is a flexible approach to design an effective large area or small area electrification system. This study demonstrated that these systems can be implemented on any single building or house or across an entire region. It is also an important technique for future electrification design. Hao.et al. [10] worked on transactive control of commercial buildings for DR, which is a form of distributed control mechanism. It is about the strategy of using market mechanism to establish self-implemented responsive load in order to maintain the power balance in electrical power grid. Which is done by using an approach of transactive control of commercial building's heating, ventilation, and air-conditioning approach (HVAC) system to achieve require results i.e. DR. System modeling and identification is presented by using system engineering building (SEB) measurements. It is showed in this study that the approach of transactive control used, worked effectively at peak shaving, load shifting and strategic conservation. However, tariffs variations need further consideration.

Akhter.et al. [11] compares two of scenarios which are used to prioritize transactive energy buyers. It is done by allowing neighborhood energy transaction in residential microgrid. At first, this energy transaction is taken according to the information collected from energy shortage of different houses. Priority of these houses depends on the energy requirements and the installed equipment like photovoltaic (PV) units or both PV and BESSs. The collected results showed in this paper described that there is not enough flexibility for energy transaction, but seller's earning rate can be significantly high. Even it is not guaranteed because of the static behavior of the transaction. Secondly, the techniques used in this paper encourage both user and the seller to increase their utilities by prioritizing the dynamic pricing method in which everyone wants to win. This technique allows the participants to become a part of the bidding of the electricity prices like other markets which also makes it more flexible. However, DSO perspective needs further consideration.

Sijie.et al. [12] proposes a new methodology of TE that allows consumers to put their requirements and demands according to the situation of power supply and reliability of enhanced power system and its economic operation. Design of demand response programs are also discussed in this paper. Which shows how to be categorized consumer's behavior with respect to a time changing tariff, and how to establish a time varying tariff that completely explains users' demand for potential response. However, transactive energy needs to be taken as a challenging solution which is expected to manage the dynamic balance and changeable demand of the supply by ensuring real time transaction between distributed energy generation and lead resources.

Koen.et al. [13] presents integrated efficient DERs with TE. Its main objective depends on the vision of smart grid, which is coordination mechanisms. In this mechanism, a large number of passively connected devices behave like actively connected to the systems and work as a local coordinating task. In this paper two key points are identified for transactive energy i.e. for transactive operational decision approach, the main operational parameters are the values which are taken from the information captured from the result of transactions between the participants. The other point is that this approach is suitable for entire infrastructure of the electrical system. However, interconnected mechanisms like loop of meshed distribution systems needs further exploration.

The most challenging part for smart grid is to co-ordinate with the increasing amount of the intelligent devices, according to the perspective of their own objectives, to make a resilient, secure, and flexible system. The analyzed result demonstrated that the overall feeder load, for reliable and economical benefit, can be regulated by the continuous change of the market-based signal. It is also described that there is an important step which allow user interfaces and program designs to work. This step is automation, which should not be expensive, and which should be easy to install and manage. M. Prabowathi. et al. [14] has researched on a competitive environmental power bidding model which was created because of reconstructing of electricity market. In such market, a power exchanging operating pool has been implemented to get the offers from the competitive suppliers with respect to the bids of the consumers. However, in this type of openly accessible environment, the introduction of TE strategy of biding in various percentages is one of the aimed achievements for electricity participants to increase their profit.

A mathematical framework is introduced in this paper to create a bidding methodology for the sellers and buyers in this regenerated electricity market. It is done by assuming that each participant gives a submission of few blocks of the real power quantities along with their bidding prices. Lei.et al. [15] discussed a problem which is stated by the distributed generation integration and the solution to that problem is microgrid power local consumption. Even rated power transaction of microgrid electricity is not enough to manage and simplify the labor cost, which is quite high. According to this study the power market can be introduced to resolve the issue of local consumption of MG. That is the reason of using block chain technology in this paper. It can also simplify and realize the point to point power and microgrid power transaction. However, cost benefit analysis for utility, energy purchaser and energy seller in the system needs further investigation.

This blockchain methodology is also implemented to facilitate the point to point electricity sales in MG. Jing.et al. [16] worked on the study of two staged optimal scheduling mode in VPP form, for DERs. Which participate in day-ahead (DA) and real-time (RT) markets. In phase 1, the VPP improves its hourly scheduled technique to increase the total gain in DA market. In phase 2, the VPP decrease the variance cost in relation to the predicted errors in the RT balancing market. According to this research, it is determined that VPP can easily make place for their scheduling strategy with respect to the expected level of risk and If a strict methodology is acquired, the probability of using DG and to trade electricity from the grid, is maximized. So, in such case, less energy trading takes place in RT market. However, from the perspective of TE, per kWh cost analysis and utilization of DG and REGs with reference to efficiency needs further examination.

The most challenging part for smart grid is to co-ordinate with the increasing amount of the intelligent devices, according to the perspective of their own objectives, to make a resilient, secure, and flexible system. The analyzed result demonstrated that the overall feeder load, for reliable and economical benefit, can be regulated by the continuous change of the market-based signal [17,18]. It is also described that there is an important step which allow user interfaces and program designs to work. This step is automation, which should not be expensive, and which should be easy to install and manage. It is also observed from the estimated result that the coordinated scheduling can efficiently neutralized the wind power function and reduce the influence of unpredictability. With the help of two-level scheduling, the risk subjection can be reduced. It is also concluded in this paper that two-level scheduling is a ductile risk-obstructing tool that can be associated with an optimal and appropriate optimal ideal plan [19]. However, impact of load variations needs to be addressed with changing tariffs in favor of either DSO or prosumer.

Research work discussed above take into account different aspects of transactive energy like peer-to-peer under blockchain regime [20]. There is huge work that has been done for the development of transactive energy system inside a house or for an electrification system. Two-way scheduling is done for various systems for day a head scheduling and real time scheduling in transactive energy and different techniques are used to engage both sellers and buyers in the system [21]. A commendable work is done in Ref. [22] regarding the planning profit and reliability results for different DR. However, the findings are subjected to addition of new feeders in radial configuration of distribution network. Moreover, deferral of new reinforcements needs to be addressed across a planning horizon. In addition, TE for the use of integrated energy systems profitably is a viable option that needs to be accessed and evaluated across meaningful indices.

Utilizing a TE based energy management technique to directly coordinate the local energy supply and demand is a promising approach for supply-demand coordination. DERs, which make it possible for prosumers, consumers, and distribution-level energy providers to exchange energy through a transactive energy system trading platform. This study includes a detailed classification from several viewpoints, including respective participants, structure, commodities, clearing method, and solution algorithm, and provides a thorough review of a transactive energy system trading [23,24]. A reputation index based framework on long-term TE history has proposed in Ref. [25] that enforces energy suppliers to honor their generation commitments and prevents them from greedy behavior in the market. A quadratically constrained programing based programming based planning framework is proposed for profit on investments by determining the installation year of new distribution feeders and energy resources, distributed energy resource (DER) placements and sizes considered by corresponding DSOs. However, impact on each prosumer and scheduling from one day to planning across large planning horizon needs further investigations.

However, despite the work done so far in transactive field, there are some gaps where the performance of the electrical system where transactive energy being implemented has to be done [[7], [8], [9], [10], [11], [12], [13], [14], [15], [16], [17], [18], [19], [20], [21], [22], [23], [24], [25], [26]]. The effect of creating an electrical market for the introduction of transactive energy on system performance considering the consumers and sellers on the voltage profile and system power losses needs to addressed. Although the optimization of resources has considered in detail across reported literature, however, post installation DGs as per “fit and forget approach” needs to be addressed from the perspective of TE and resources optimization. Also, system performance while considering the variation of percentage of transactive energy in a same system with respect to conventional electrification system [24]. Thus, ensuring the overall benefit or drawbacks of have integrated transactive approach for distribution system. Planning perspective while developing an integrated transactive system over conventional distribution has also been missing. So, research work is done considering system performance of the period of 5 years in terms of cost benefit analysis for utility, energy purchaser and energy seller in the system, voltage profile of the system, per kWh cost analysis and utilization of distributed resources more efficiently as compared to conventional electrical system. The main aim of this work is to address the limitations and perspectives, that have left in the reported literature and propose a solid framework for the future distribution mechanisms considering the utility (DSO) relevance along with prosumer comfort from the viewpoint of economic and technical sides of the services. The limitations of the research gaps have illustrated in the appendix section in Table A1.

In this paper, the proposed approach is to design a TE framework and evaluated across IEEE 69-bus meshed MG test system from the perspective of scheduling and planning. In the performed research comparison of system with and without transactive energy by considering line loses, voltage profile and cost benefit to the utility and consumers have been done. It consists of energy management system, prosumers and consumers. Prosumers in the designed system have both conventional and non-conventional resources for generation. The contributions in this paper includes the following highlights.

• Develop traditional IEEE 69 bus system and convert into interconnected system
• Involve prosumers to develop transactive system across three cases of TE at 10%, 15% and 20%.
• Maximize profit for producer and consumers
• Minimize energy imbalance by efficiently using DERs
• Transactive energy for the planning perspective to analyze cost
• Reduction in total production cost of energy units
• Reduction in active power losses
• Transactive energy impact on voltage profiles
• Evaluation of proposed approach across planning horizon of five years.
• Coordinated distribution system planning considering transactive distribution system operator (DSO) based market.
• The post installation DGs as per “fit and forget approach” have addressed from perspective of TE and resources optimization.

The paper is arranged as follows. The methodology is discussed in Section 2 encapsulating simulation test setup, load modelling, prosumer modelling, proposed approach, optimization algorithm and scheduling problem formulation. In Section 3, the performance evaluation is evaluated across prosumers across various percentages of transaction energy. The results and discussions are presented in Section 4. The paper is concluded in Section 5.

## 2 Methodology

The Methodology includes various sections as follows.

### 2.1 Simulation test setup

**Fig. 1:**
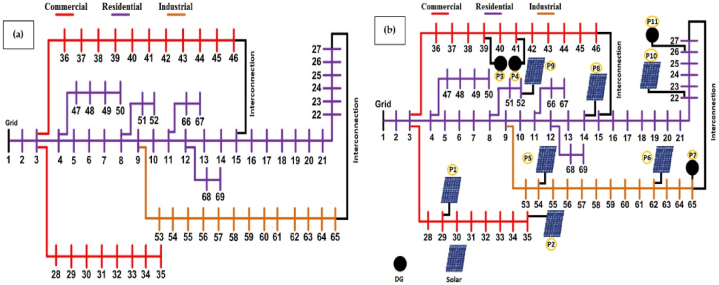
(a) IEEE 69 meshed microgrid distribution system without DG, (b) with prosumers including DGs.

The test system for the observation of transactive energy impact on the system is described on IEEE 69 bus system multi-loop configured meshed distribution network, is developed for the observation of transactive energy impact over the period of 5 years. Bus system is developed in OpenDSS and simulations are being carried out in Python 3.7. To present the transactive system impact, variable load over the period of 24 h has been taken in this model and then scheduling is done. Comparison between non-transactive system with the transactive applied system is done over the period of one day with the time interval of 1 h. To observe the impact of transactive approach over the period of 5 years, 7.5% per year load growth has been adopted. IEEE 69 mesh system without any prosumers is compared with (i) 10% transactive, (ii) 15% transactive and (iii) 20% transactive in the system. IEEE 69 mesh system is taken as base case scenario and line loses, demand reduction, voltage profile and cost of the system is compared with the suggested different transactive approach applied in the same system. The test IEEE 69 distribution bus system with mesh topology is constructed in OpenDSS with three types of loads namely, residential load, industrial load and commercial load. To observe the hourly consumption, real power losses, cost fluctuation and changing voltage profile, time interval of 1 h is considered while developing the system. The electrical power is delivered from utility generation only. Test system has both real and reactive power acting as load. Thus, as the load at specific interval increases, line losses and voltage drop more as compare to the less load interval. Scheduling has been done for the real power only. Real power from all types of loads are aggregated and with that line losses are calculated for the specific interval and then fed to the system operator for the scheduling process. The commercial, residential and industrial loads are illustrated with respective colors in this paper with 11 prosumers, pre-installed with 7 PV based system without storage and 4 diesel generator-based systems. The meshed configured MG distribution test system is shown in Fig. 1 (a). Whereas nodes with DGs (prosumers) are shown at predetermined locations as shown in Fig. 1(b).

### 2.2 Load modelling

**Fig. 2:**
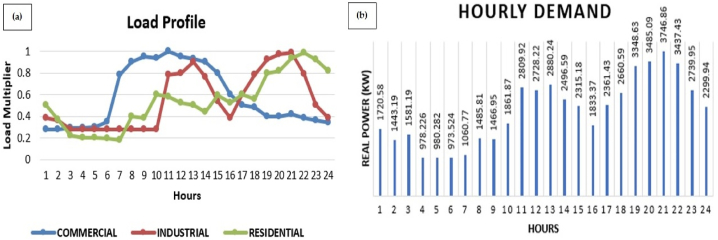
(a) Daily Load Pattern, (b) Aggregated load.

To perform the scheduling for a whole day to observe day a head market, load is modeling for whole day with a time interval of 1 h. Load is divided into three types of load namely residential load, industrial load and commercial load. Daily load is normalized such as the peak demand is at “1” (i.e. 100%). Time varying demand pattern of residential, industrial and commercial load over a time period of 24 h is shown in Fig. 2 (a). For scheduling purposes hourly demand from all the three types of loads are aggregated into a bulk load and then the load data is given to the optimizer. Aggregated load of base case is shown in Fig. 2 (b).

### 2.3 Prosumer modelling

**Fig. 3:**
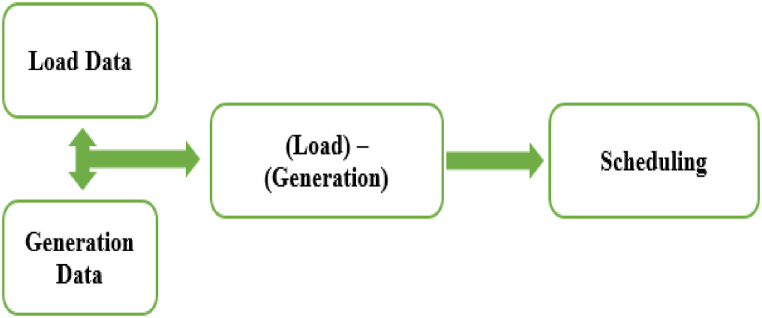
Prosumer Modelling and functioning.

Fig. 3 shows the functioning of prosumers where prosumers are modeled for transactive system are of two types. First has solar system installed for the generation and second has diesel generator for the generation. Prosumers act as an active component in the system providing the data for the load and generation at the time. Hourly load and generation data are feed into the system and then calculated for the action of prosumer as consumer or producer. If load is greater than the generation prosumer acts as a consumer and if generation is greater than the load, prosumer acts as a producer.

### 2.4 Solar system modelling

PV array output depends upon various environmental factors for example solar irradiance. To calculate the amount of energy absorbed by the solar cell, solar irradiance value at the time should be known. By applying the open circuit condition to the equation of solar cell given in eqns. (1), (2)):

(1) $$I=I_{sc}-I_{0}\;\left(e^{\frac{V_{OC}}{V_{T}}}-1\right),I=0,for\;open\;circuit$$

Open circuit voltage is given by:

(2) $$V=V_{T}\;\ln\;\left(1+\frac{I_{sc}}{I_{0}}\right)$$

Above equations indicate that effect of the irradiance is much larger in the short circuit current than in the open circuit voltage. The total energy absorbed by the PV cell is given by the following eqn. (3):

(3) $$E_{c}=\rho \sigma_{c}\tau_{g}G\left(t\right)$$

where:

“G(t)” is value of solar irradiance.

$\prime \prime \rho^{\prime \prime }$ is the cell packing factor and is defined as the ratio of area of solar cell to the area of blank absorber. The PV panel has been set up at the clear are. It is mean that PV cell can absorb 100% of solar irradiance. So, the cell packing factor is assumed to be 1.

$\prime \prime {\sigma_{c}}^{\prime \prime }$ is cell absorptivity to sunlight.

${{\prime \prime \tau }_{g}}^{\prime \prime }$ is a fraction transmitted through the front glass and for this study, low iron glass was used which is equal to 0.95.

Solar energy used in transactive approach is feed to the grid with the help of inverter which converts DC current into AC current. Inverter efficiency and other AC and DC losses of solar system are also considered while developing the system. Solar system for the prosumer is designed in Helioscope (an online facility to develop solar system) to get the solar generation over a year.

**Fig. A1:**
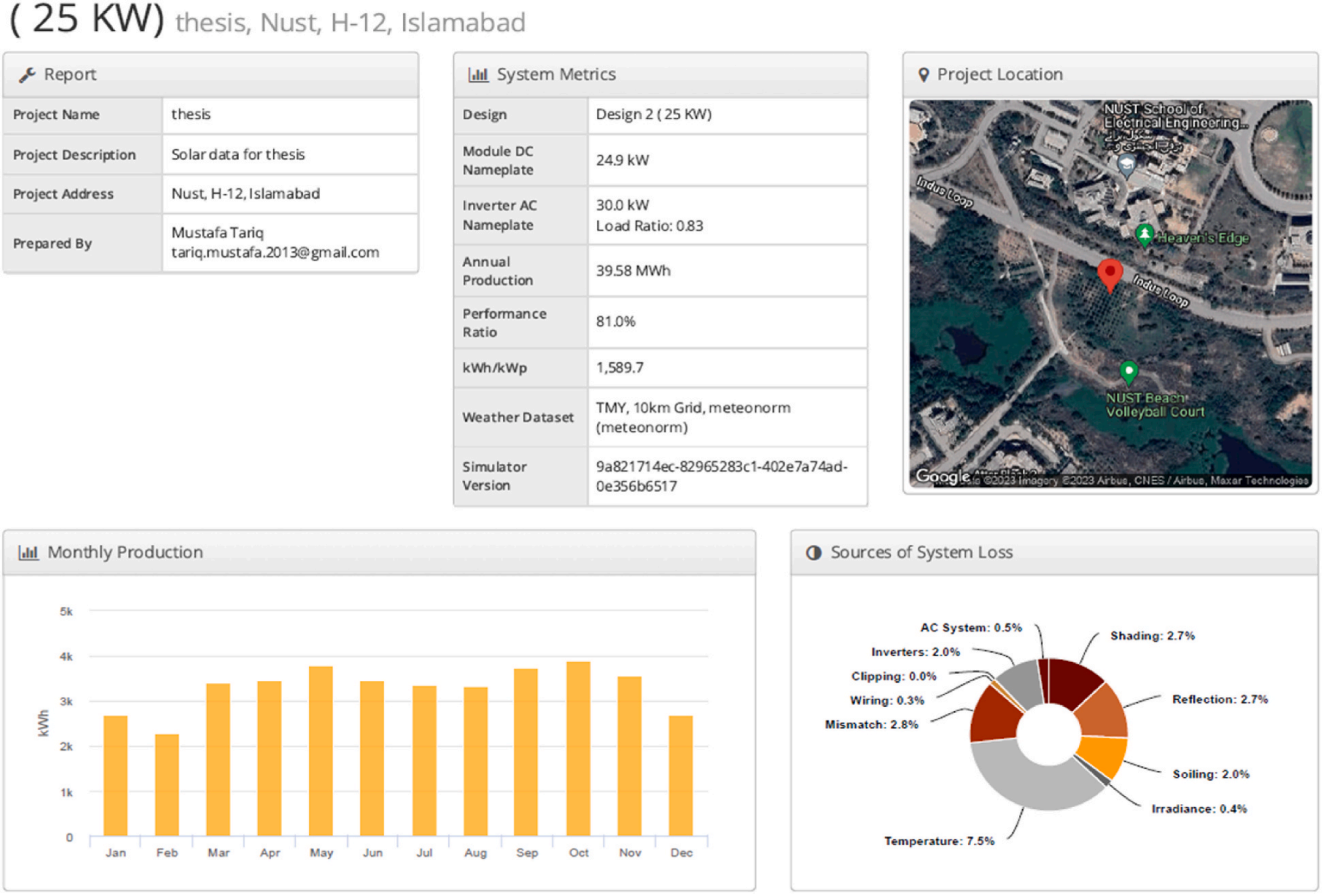
25 kW Solar system design

After getting the CSV file from the Helioscope, solar data of 24 h has been taken to perform the load scheduling. For Example, design of single prosumer having 25 kW solar system is shown in figure A1 in the appendix Section.

### 2.5 Proposed approach and optimization algorithm

**Fig. 4:**
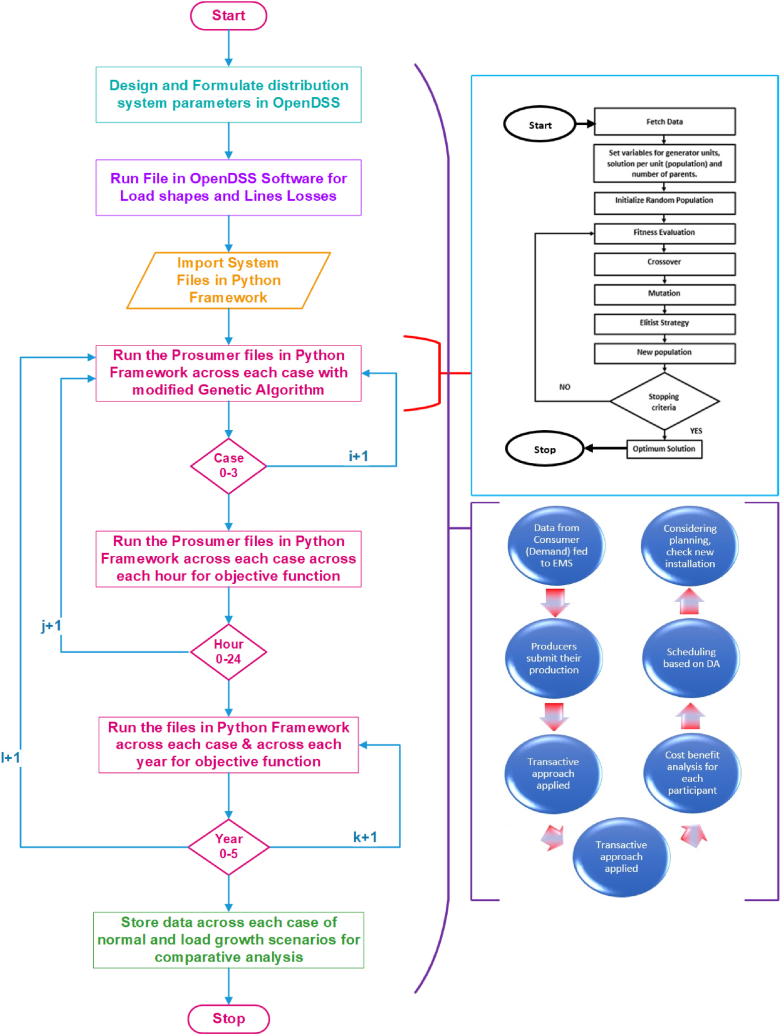
Methodology for TE and Flow chart of proposed approach with Genetic Algorithm.

For optimization, one of the adaptive heuristic search algorithms named Genetic Algorithm (GA) is used [25]. The GA is constructed on natural selection and genetics. Genetic algorithms are usually used to obtain high quality optimization that are near to the global optima. Natural selection in the genetic algorithm means the genes that the fittest for the further progress are selected to regeneration. Genetic algorithm can also be called “Survival of the fittest” among generations for solving a required objective problem. Flowchart of and transactive energy approach applied genetic algorithm is given in Fig. 4.

The proposed approach is shown as follows.

1. All the design, line, load and distribution parameters are made in OpenDSS software.
2. The design file is run aiming at achieving load shapes and line losses.
3. Then the file is imported in Python Framework, where prosumer files (considering prosumer number and picking the consumer type) are run repeatedly using modified generic algorithm.

{ *Each generation consists of population of individuals thus providing the search space and possible solution among the individuals. Each individual can also be regarded as chromosome. Implementation of GA can be expressed as*.

• *Generate initial random population of chromosomes.*
• *Evaluate the fitness of each chromosome in the population by using objective function.*
• *Select fittest chromosomes from the population that will act as the parents of new generation.*
• *Breed the parent chromosomes by performing crossover.*

• *Add Mutation in the children chromosomes.*
• *Replace the older generation with the new generation.*
• *Evaluate for the stopping criteria. If the stopping criteria is reached that is best solution chromosomes for the problem is found, output the best chromosomes as optimal solution.*

*Otherwise go to the Evaluation step and repeat the process until best solution is found.*}

4. The prosumer files are run multiple times across each case (base case 0% TE and three cases 10%, 15% and 20%) of TE penetration.
5. The objective functions like minimizing cost (unit and total production cost) and reducing line losses are evaluated to the converged value.
6. Then across each case, the program is run across each hour of scheduling horizon until converged.
7. Then across each case, the program is run across each year till 5th year of planning horizon until converged.
8. Finally, the results are saved along with comparative analysis.

### 2.6 Scheduling problem formulation and objective function

Initially load is calculated for each appliance for individual house. Then simply added all appliances load for individual house. Then we calculated summer and winter loads using eq. (2) and eq. (3) respectively. Then yearly load for each house is calculated using eq. (4). Then we took average of all houses yearly loads using eq. (5) and estimated per house average load using eq. (6). Finally, the entire load is calculated using eq. (7). Mathematical formulas derived for load estimation are given below. The cost function is mathematically expressed as shown in eqn. (4):

(4) Minimize:F(x,u)S.t.G(x,u)=0;H(x,u)≤0

Where.

*F* = Cost Function

*x* = Vector of dependent variable

*u* = dependent of control variable.

*G* (*x*, *u*) = Set of non-linear equality constraints.

*H* (*x*, *u*) = Set of non-linear inequality constraint.

Objective function for the scheduling problem is given by eqn. (5):

(5) $$\mathrm{Min}:F_{t}=\sum_{t=1}^{T}\left(\sum_{i=1}^{N}F_{it}\right)=\sum_{t=1}^{T}\left(\sum_{i=1}^{Ng}\left(a_{it}+b_{it}P_{git}+c_{it}P_{git}^{2}\right)+\sum_{i=1}^{NDg}\left(a_{it}+b_{it}P_{Dgit}+c_{it}P_{Dgit}^{2}\right)+\sum_{i=1}^{NDr}\left(a_{it}+b_{it}P_{Drit}+c_{it}P_{Drit}^{2}\right)\right)$$

Where.

$F_{t}$ = Total generation cost;

$F_{i}$ = Generation cost of unit i;

N = Total number of power units;

Ng = Total number of Utility generation units;

NDg = Total number of Distributed Generation units;

NDr = Total number of Distributed Renewable units;

$a_{i}$, $b_{i}$, $c_{i}$ = Cost coefficient of unit i;

$P_{gi}$ = Power output of Utility generation unit i;

$P_{DGi}$ = Power output of Distribution Generation unit i;

$P_{DRi}$ = Power output of Distribution Renewable unit i;

Supply Demand Balance Constraint is shown in eqn. (6):

(6) $$\sum_{t=1}^{T}\left(\sum_{i=1}^{Ng}P_{git}+\sum_{i=1}^{NDg}P_{Dgi}+\sum_{i=1}^{NDr}P_{Drit}\;\right)=\sum_{t=1}^{T}\left(P_{Dt}+P_{Lt}\right)$$

where.

$P_{D}$ = Power demand;

$P_{L}$ = Distribution power loss;

Power Limit Constraints:

(7) $$P_{gi}^{\min}\leq P_{g}\leq P_{gi}^{\max}\;;\;P_{Dgi}^{\min}\leq P_{Dg}\leq P_{Dgi}^{\max}\;;\;P_{Dri}^{\min}\leq P_{Dr}\leq P_{Dri}^{\max}$$

in power system, utility generation units are connected to distribution network via power transmission network. Scheduling is performed depending upon the following factors such as.

(i) Generation cost of all the connected units
(ii) Aggregated power demand and line losses
(iii) Generator limits

Objective function shown in (5) provides an optimal real power flow which focuses on reducing the operating cost. Whereas, (6) shows the supply-demand constraint considering the utility generation, distributed generation and distributed renewable energy while considering the losses and (7) indicate the generators upper and lower limit constraint.

## 3 Performance evaluation across prosumers across various percentages of transaction energy

The research presents an integrated transactive energy market system that ensures the involvement of both production and consumer side. Two-way profit can be obtained by involving consumer that can sell extra energy from DERs in addition to utility generation. Thus, utility and consumers get benefit by utilizing cheap and decentralized electricity more efficiently. First of all, profit has to maximized for all the participants in DA market by economic dispatch. The daily benefits of prosumers (participants of the system) and to utility can-be achieved utilizing decentralized energy. Utility can provide same amount of energy with less losses and also at lesser cost as compare to conventional distribution system. Secondly transactive impact in a long-term planning of electrical systems involving DERs needs to be discussed. In planning, daily benefit of the consumers decreases due to load growth, however, the overall system benefit is seen as compared to the expensive energy costs in conventional systems. The impact of transactive approach over the period of 5 years, 7.5% per year load growth has been adopted. IEEE 69 mesh system without any prosumers is compared with. Table 1, Table 2, Table 3 and Table 4 shows load data estimation for all the rural communities.

• Three cases across 10%; 15% and 20% transactive energy, respectively;
• IEEE 69 mesh system is taken as base case scenario;
• Line loses, demand reduction, voltage profile and cost of the system is compared with the suggested different transactive approach applied in the same system.
• Evaluations of case-1 (10% TE) will be carried out across 5-year planning horizon.

**Table-1:** Prosumers classification with 10% Transactive energy.

S#	Prosumer #	Bus #	Source Type	Load Type	Cap (kW)	US'/kWh	$/kWh
1	P1	29	Solar PV	Commercial	60	6.5	0.065
2	P2	35	Solar PV	Commercial	32	5.1	0.051
3	P3	39	Diesel Generator	Commercial	50	4	0.04
4	P4	41	Diesel Generator	Commercial	30	6.8	0.068
5	P5	54	Solar PV	Industrial	30	7	0.07
6	P6	62	Solar PV	Industrial	40	4.5	0.045
7	P7	65	Diesel Generator	Industrial	50	6.2	0.062
8	P8	14	Solar PV	Residential	25	5.15	0.0515
9	P9	52	Solar PV	Residential	20	9	0.09
10	P10	22	Solar PV	Residential	20	8.5	0.085
11	P11	26	Diesel Generator	Residential	45	7.6	0.076
Utility Power Supply from Substation (Maximum Capacity)					6000	11.64	0.1164

**Table-2:** Prosumers classification for 15% Transactive energy.

S#	Prosumer #	Bus #	Source Type	Load Type	Cap (kW)	US'/kWh
1	P1	29	Solar PV	Commercial	100	6.5
2	P2	35	Solar PV	Commercial	50	5.1
3	P3	39	Diesel Generator	Commercial	60	4
4	P4	41	Diesel Generator	Commercial	50	6.8
5	P5	54	Solar PV	Industrial	40	7
6	P6	62	Solar PV	Industrial	60	4.5
7	P7	65	Diesel Generator	Industrial	70	6.2
8	P8	14	Solar PV	Residential	35	5.15
9	P9	52	Solar PV	Residential	30	9
10	P10	22	Solar PV	Residential	30	8.5
11	P11	26	Diesel Generator	Residential	65	7.6
Utility Power Supply from Substation (Maximum Capacity)					6000	11.64

**Table-3:** Prosumers classification for 20% Transactive energy.

S#	Prosumer #	Bus #	Source Type	Load Type	Cap (kW)	US'/kWh
1	P1	29	Solar PV	Commercial	120	6.5
2	P2	35	Solar PV	Commercial	70	5.1
3	P3	39	Diesel Generator	Commercial	70	4
4	P4	41	Diesel Generator	Commercial	60	6.8
5	P5	54	Solar PV	Industrial	50	7
6	P6	62	Solar PV	Industrial	80	4.5
7	P7	65	Diesel Generator	Industrial	100	6.2
8	P8	14	Solar PV	Residential	50	5.15
9	P9	52	Solar PV	Residential	40	9
10	P10	22	Solar PV	Residential	40	8.5
11	P11	26	Diesel Generator	Residential	80	7.6
Utility Power Supply from Substation (Maximum Capacity)					6000	11.64

**Table 4:** Energy Cost in Conventional Distribution system with 0% TE for base year.

S#	Load at Bus #	kW load per day	Purchase Cost per day (US$/kWh)
1	Load at bus 29	359.32	42.7414
2	Load at bus 35	82.92	9.8634
3	Load at bus 39	331.68	39.4536
4	Load at bus 41	16.58	1.9726
5	Load at bus 54	345.048	41.2127
6	Load at bus 62	418.24	49.9548
7	Load at bus 65	771.13	92.1043
8	Load at bus 14	102.72	12.2495
9	Load at bus 52	46.224	5.51231
10	Load at bus 22	64.2	7.6559
11	Load at bus 26	179.76	2143.67
Net Total:		2717.838	313.15721

### 3.1 Case-1: meshed IEEE 69 bus MG distribution system with 10% transactive energy

In 10% transactive energy approach 402 kW of generation is connected through 11 prosumers. 56.46% of the transactive energy is provided by solar energy and rest is provided by diesel generator. Solar systems are designed in Helioscope and data for the hourly production is taken from the datasheet of the simulated model. NUST, H-12, Islamabad is chosen as the data subjected to location for the solar system for all the solar energy systems added in IEEE 69 bus system.

• In 10% transactive energy approach, 402 kW of generation is connected through 11 prosumers. The prosumer classification and scheduling for initial year is given in Table-1.
• 56.46% of the transactive energy is provided by solar energy and rest is provided by diesel generator.
• Solar systems are designed in Helioscope and data for the hourly production is taken from the datasheet of the simulated model.
• NUST, H-12, Islamabad is chosen as the location for the solar system for all the solar energy systems added in IEEE 69 bus system.
• The evaluations of case-1 (10% TE) will be carried out across 5-year planning horizon.

### 3.2 Case-2: meshed IEEE 69 bus MG distribution system with 15% transactive energy

In 15% transactive energy approach 590 kW of generation is connected through 11 prosumers.

• 58.4% of the transactive energy is provided by solar energy and rest is provided by diesel generator. The prosumer classification and scheduling for 15% TE is given in Table-2.
• Solar systems are designed in Helioscope and data for the hourly production is taken from the datasheet of the simulated model.
• NUST, H-12, Islamabad is chosen as the location for the solar system for all the solar energy systems added in IEEE 69 bus system.
• The evaluations of case-2 (15% TE) will be carried out across 5-year planning horizon.

### 3.3 Case-3: meshed IEEE 69 bus MG distribution system with 20% transactive energy

In 20% transactive energy approach 760 kW of generation is connected through 11 prosumers.

• 59.2% of the transactive energy is provided by solar energy and rest is provided by diesel generator. The prosumer classification and scheduling for 20% TE is given in Table-3.
• Solar systems are designed in Helioscope and data for the hourly production is taken from the datasheet of the simulated model.
• NUST, H-12, Islamabad is chosen as the location for the solar system for all the solar energy systems added in IEEE 69 bus system.
• The evaluations of case-3 (20% TE) will be carried out across 5-year planning horizon.

## 4 Results and discussions

For planning perspective, the comparison is made between distribution systems with simple mesh distribution system using only utility as generation source and across three cases with 10%, 15% and 20% TE with prosumers. The evaluation is initially carried across same load and line impedance over the period of five years. The evaluation parameters include voltage index, real power line losses and per kWh cost.

### 4.1 Base case evaluation and Year-0 evaluation with 10% TE

**Fig. 5:**
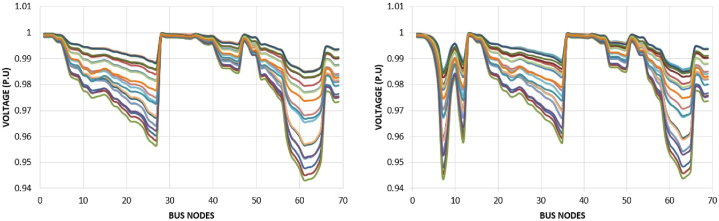
Voltage profile of Test System in year 0 (a) Without TE; (b) with 10% TE.

The voltage profile of test distribution system without and with 10% TE is shown in Fig. 5 (a) and (b), across 24 h of 05 June 2019 correlated with NUST data, respectively. It can be observed that as the load of the consumer increases, voltage of the weakest node further decreases. Where each color represents an hour and shows the voltage index during time span of 24 h.

**Fig. 6:**
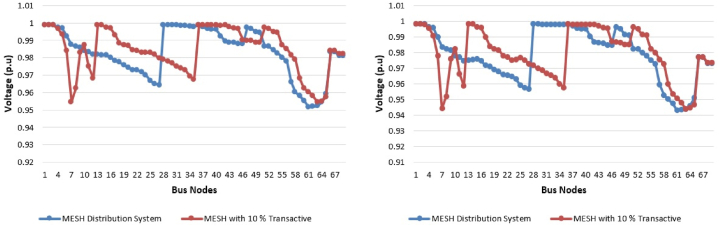
Voltage profile 12th hour and at 21st hour in year 0 (a) Without TE; (b) with 10% TE.

**Fig. 7:**
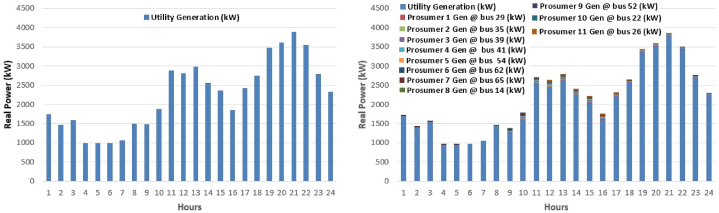
Generators contribution across 24 h in year 0 (a) Without TE; (b) with 10% TE.

The prosumers with PV based distribution generation only provides the real power in the system and reactive power is provided by utility generation in proposed approach. The comparison for the voltage profile at 12th hour and at 21st hour is illustrated in Fig. 6, where in 12th hour is where maximum input from PV is present and 21st hour give maximum load scenario. It can be seen that system with 10% TE has high voltage at peak as compare to the normal mesh distribution network. Generators participating in the scheduling are aforementioned given in table-1. For the scheduling of system without TE, as shown in Fig. 6(a), only utility generation is used and for the transactive case utility generation with 11 prosumers are participating in the scheduling, as in Fig. 6(b). The cost analysis is performed after performing scheduling by genetic algorithm without (0% TE) and with 10% TE. In 0% TE, only generation point is utility generation and in 10% TE, all the participants take part in the electricity market. As aforementioned in Table 1, eleven prosumers are given that will take part along with the utility generation in the market. The generators contribution of utility in Fig. 7(a), and by prosumers in Fig. 7(b)–is shown over the period of 24 h of 23 September 2019 correlated with NUST data.

**Fig. 8:**
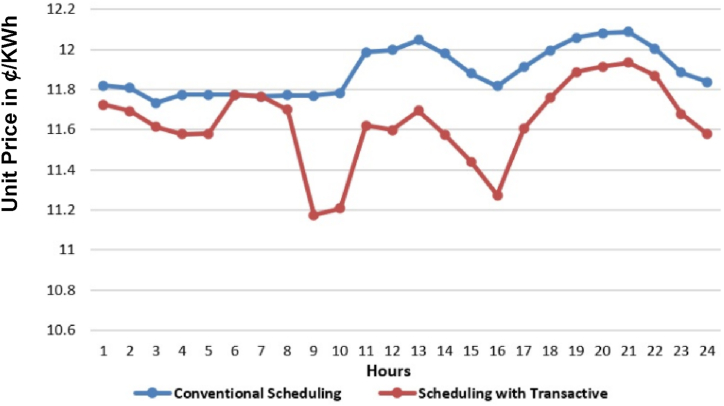
Cost comparison in ¢/kWh (24 h) in year 0 (a) Without TE; (b) with 10% TE.

Fig. 8 shows the cost per kWh energy over the period of 24 h. The “generation cost” is the actual cost of generation unit for producing 1 kWh energy that accounts for demand and loss accumulatively. While “demand cost” is the cost of producing 1 kWh energy as per demand of consumer. Fig. 8 shows two data lines, one in Fig. 8(a)–is the cost of per kWh for the general scheduling without involvement of any prosumer while the other is the cost of scheduling with the involvement of prosumers. It is visible that system with transactive energy has lower per kWh cost for the same amount of energy with respect to the conventional scheduling. Peak kWh cost in Fig. 8(b) for general scheduling is “US'. 12.0867” and for system with TE is “US'. 11.9351” at 21st hour. After the scheduling of whole day, the total electricity cost for day of MDS without transactive energy is “US$. 6286.852” and total electricity cost for day of MDS with transactive energy is “US$. 5963.499”. This shows a difference of “US$. 323.353” less amount in facilitating the same quantity of customers with same demand in MDS with 10% TE from MDS in comparison with 0% TE, respectively.

**Fig. 9:**
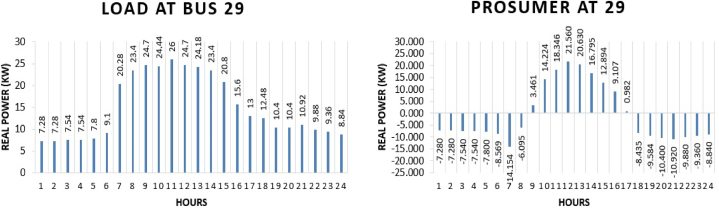
Hourly real power of prosumer-1 (@Bus 29) in MDS (a) 0% TE; (b) with 10% TE.

The core aim of TE is to involve customers to get benefit of low-cost electricity and distributed generation utility. Customer's involvement occurs if prosumers find some benefit having a partnership in this system. Prosumer 1 is present at bus 29 of IEEE 69 bus system. Customer benefit by involving in transactive approach can be seen in the comparison of electricity purchase and sell in system with transactive approach and without transactive approach. Firstly, there is a huge change in the demand pattern of the customer as it is acting as a prosumer rather than traditional customer. These changes can be seen in Fig. 9 (a)-(b).

Fig. 9 (a) represents the customer without any generation capability that is only acting as load in the system while Fig. 9 (b) represents the same customer with solar system installed and is acting as a prosumer in transactive approach. In Fig. 9 (a), all data values are in positive which shows the hourly real power requirement of the customer in kWh while in Fig. 9 (b), data values are in positive and negative. Positive data values in Fig. 9 (a) are the solar output that the prosumer can provide to the market after covering its own load and negative values are the load demand of the prosumer at that particular hour. Total energy required for the whole day in conventional system is “359.32 kWh” while in system with the transactive energy “343.64 kWh” energy is produced by the solar system and “115.25 kWh” is being used for the scheduling and the remaining is used to fulfil personal requirements. Benefit of the prosumer in TE can be seen when the cost of scheduling of the day of conventional system and transactive system is compared. The cost of purchasing energy in conventional system for the whole day of load at bus 29 is “US$. 42.74141”. The cost of purchasing energy for the whole day with transactive approach is “US$. 15.70707” and cost of selling as a prosumer is “US$. 7.49184”. So, the total expense of prosumer 1 for the whole day is “US$. 8.21523”. This shows a huge cost difference of “US$. 34.5261” in purchase energy for running the same amount of load for the whole day.

**Fig. 10:**
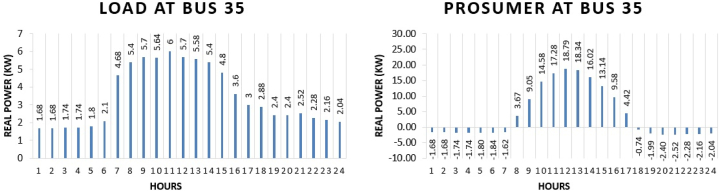
Hourly real power of prosumer-2 (@Bus 35) in MDS (a) 0% TE; (b) with 10% TE.

The prosumer 2 is present at bus 35 of IEEE 69 bus system. Customer benefit by involving in transactive approach can be seen in the comparison of electricity purchase and sell in system with transactive approach and without transactive approach. The real power pattern of customer at bus 35 in conventional and transactive system is shown in Fig. 10 (a)-(b). Total energy required for the whole day in conventional system is “82.92 kWh” while in system with the transactive energy (TE) “181.55 kWh” energy is produced by the solar system and “122.72 kWh” is being used for the scheduling and the remaining is used to fulfil personal requirements. Benefit of the prosumer in transactive energy can be seen when the cost of scheduling of the day of conventional system and transactive system is compared. The cost of purchasing energy in conventional system for the whole day of load at bus 35 is “US$. 9.8634”. Cost of purchasing energy for the whole day with transactive approach is “US$. 3.08213” and cost of selling as a prosumer is “US$. 6.259”. So, the total benefit of prosumer 2 for the whole day is “US$. 3.1768”.

**Fig. 11:**
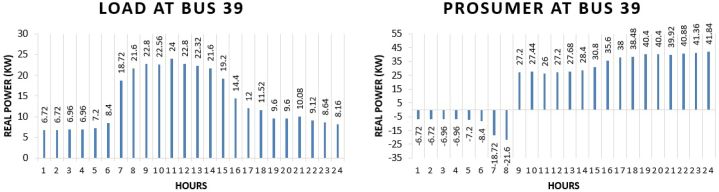
Hourly real power of prosumer-3 (@Bus 39) in MDS (a) 0% TE; (b) with 10% TE.

The real power pattern of prosumer 3 at bus 39 in conventional and TE is shown in Fig. 11 (a)-(b). Total energy required for the whole day in conventional system is “331.68 kWh” while in system with the transactive energy “800 kWh” energy is produced by the diesel generator and “544.826 kWh” is being used for the scheduling and the remaining is used to fulfil personal requirements. Benefit of the prosumer in transactive energy can be seen when the cost of scheduling of the day of conventional system and transactive system is compared. Cost of purchasing energy in conventional system for the whole day of load at bus 39 is “US$. 39.45361”. Cost of purchasing energy for the whole day with transactive approach is “US$. 9.74054” and cost of selling as a prosumer is “US$. 26.16041”. The benefit of “US$. 16.4198” is observed.

**Fig. 12:**
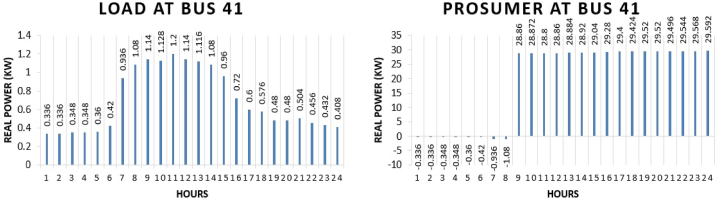
Hourly real power of prosumer-4 (@Bus 41) in MDS (a) 0% TE; (b) with 10% TE.

The real power pattern of prosumer 4 at bus 41 in conventional and TE is shown in Fig. 12 (a)-(b). Total energy required for the whole day in conventional system is “16.584 kWh” while in system with the transactive energy “480 kWh” energy is produced by the diesel generator and “461.57 kWh” is being used for the scheduling and the remaining is used to fulfil personal requirements. Benefit of the prosumer in transactive energy can be seen when the cost of scheduling of the day of conventional system and transactive system is compared. The cost of purchasing energy in conventional system for the whole day of load at bus 41 is “US$. 1.97268”. Cost of purchasing energy for the whole day with transactive approach is “US$. 0.487” and cost of selling as a prosumer is “US$. 31.38739”. So, the total benefit of prosumer 4 for the whole day is “US$. 30.90”.

**Fig. 13:**
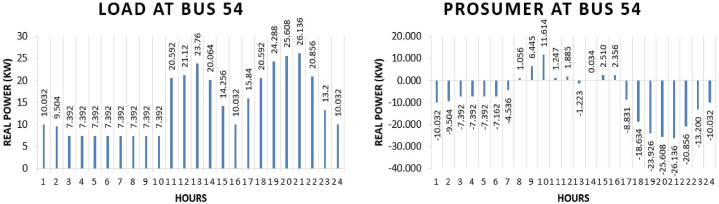
Hourly real power of prosumer-5 (@Bus 54) in MDS (a) 0% TE; (b) with 10% TE.

The real power pattern of prosumer 5 at bus 54 in conventional and TE is shown in Fig. 13 (a)-(b). Total energy required for the whole day in conventional system is “345.048 kWh”. The cost of purchasing energy in conventional system for the whole day of load at bus 54 is “US$. 41.21278”. While in system with the transactive energy “170.338 kWh” energy is produced and “22.122 kWh” is being used for the scheduling. Cost of purchasing energy is “US$. 23.6440” and cost of selling as a prosumer is “Rs 154.86”. Expense of prosumer is “US$. 22.0954”.

**Fig. 14:**
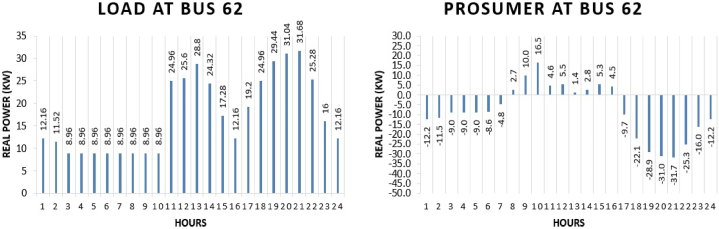
Hourly real power of prosumer-6 (@Bus 62) in MDS (a) 0% TE; (b) with 10% TE.

Prosumer 6 is present at bus 62. The real power pattern of prosumer 6 at bus 62 in conventional and TE is shown in Fig. 14 (a)-(b). Total energy required for the whole day in conventional system is “418.24 kWh” while in system with the transactive energy “230.722 kWh” energy is produced by the solar system and “50.61 kWh” is being used for the scheduling and the remaining is used to fulfil personal requirements. The cost of purchasing energy in conventional system for the whole day of load at bus 62 is “US$. 49.9548”. Cost of purchasing energy for the whole day with transactive approach is “US$. 28.385” and cost of selling as a prosumer is “US$. 2.2775”. So, the total expense of prosumer 6 for the whole day is “US$. 26.1075”. This shows a cost difference of “US$. 23.8473” in purchase energy for running the same amount of load for whole day.

**Fig. 15:**
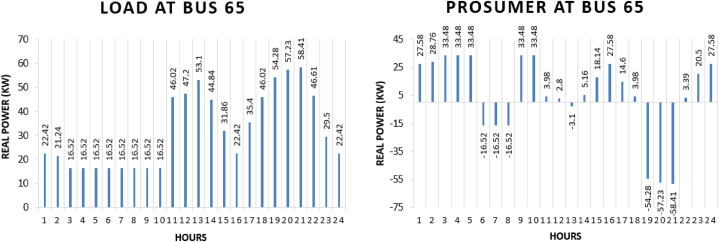
Hourly real power of prosumer-7 (@Bus 65) in MDS (a) 0% TE; (b) with 10% TE.

Prosumer 7 is present at bus 65 of IEEE 69 bus system. The real power pattern of prosumer 7 at bus 65 in conventional and TE is shown in Fig. 15 (a)-(b). Total energy required for the whole day in conventional system is “771.13 kWh” while in system with the transactive energy “900 kWh” energy is produced by the diesel generator and “345.62 kWh” is being used for the scheduling and the remaining is used to fulfil personal requirements. The cost of purchasing energy in conventional system for the whole day of load at bus 65 is “US$. 92.10432”. Cost of purchasing energy for the whole day with transactive approach is “US$. 26.4264” and cost of selling as a prosumer is “US$. 21.4285”. So, the total expense of prosumer 6 for the whole day is “US$. 4.9979”. This shows a cost difference of “US$. 87.1064” in purchase energy for running the same amount of load for the whole day.

**Fig. 16:**
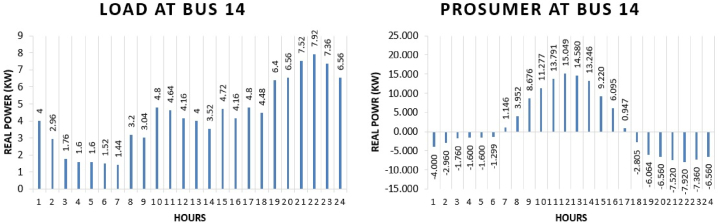
Hourly real power of prosumer-8 (@Bus 14) in MDS (a) 0% TE; (b) with 10% TE.

Prosumer 8 is present at bus 14 of IEEE 69 bus system. The real power pattern of prosumer 8 at bus 14 in conventional and TE is shown in Fig. 16 (a)-(b). Total energy required for the whole day in conventional system is “102.72 kWh” while in system with the transactive energy “142.69 kWh” energy is produced by the solar system and “94.515 kWh” is being used for the scheduling and the remaining is used to fulfil personal requirements. The cost of purchasing energy in conventional system for the whole day of load at bus 14 is “US$. 12.2496”. Cost of purchasing energy for the whole day with transactive approach is “US$. 6.832” and cost of selling as a prosumer is “US$. 4.849”. So, the total expense of prosumer 8 for the whole day is “US$. 1.9832”. This shows a cost difference of “US$. 1.027” in purchase energy for running the same amount of load for the whole day.

**Fig. 17:**
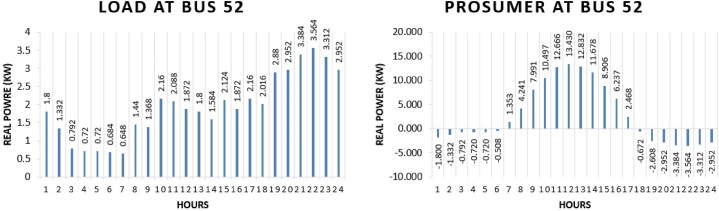
Hourly real power of prosumer-9 (@Bus 52) in MDS (a) 0% TE; (b) with 10% TE.

Prosumer 9 is present at bus 52 of IEEE 69 bus system. The real power pattern of prosumer 9 at bus 52 in conventional and TE is shown in Fig. 17 (a)-(b). Total energy required for the whole day in conventional system is “46.224 kWh” while in system with the transactive energy “113.20 kWh” energy is produced by the solar system and “89.02 kWh” is being used for the scheduling and the remaining is used to fulfil personal requirements. The cost of purchasing energy in conventional system for the whole day of load at bus 52 is “US$. 5.5123”. Cost of purchasing energy for the whole day with transactive approach is “US$. 2.9817” and cost of selling as a prosumer is “US$. 6.1428”. So, the total benefit of prosumer 8 for the whole day is “US$. 3.1611”.

**Fig. 18:**
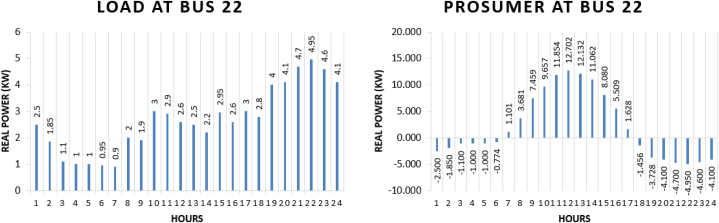
Hourly real power of prosumer-10 (@Bus 22) in MDS (a) 0% TE; (b) with 10% TE.

Prosumer 10 is present at bus 22 of IEEE 69 bus system. The real power pattern of prosumer 10 at bus 22 in conventional and TE is shown in Fig. 18 (a)-(b). Total energy required for the whole day in conventional system is “64.2 kWh” while in system with the transactive energy “113.20 kWh” energy is produced by the solar system and “80.48 kWh” is being used for the scheduling and the remaining is used to fulfil personal requirements. The cost of purchasing energy in conventional system for the whole day of load at bus 22 is “US$. 7.6559”. Cost of purchasing energy for the whole day with transactive approach is “US$. 4.2232” and cost of selling as a prosumer is “US$. 6.0364”. So, the total benefit of prosumer 8 for the whole day is “US$. 1.8132”.

**Fig. 19:**
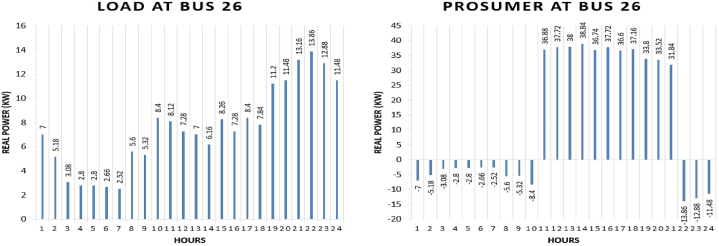
Hourly real power of prosumer-11 (@Bus 26) in MDS (a) 0% TE; (b) with 10% TE.

Prosumer 11 is present at bus 26 of IEEE 69 bus system. The real power pattern of prosumer 11 at bus 26 in conventional and TE is shown in Fig. 19 (a)-(b). Total energy required for the whole day in conventional system is “179.76 kWh” while in system with the transactive energy “495 kWh” energy is produced by the diesel generator and “394.33 kWh” is being used for the scheduling and the remaining is used to fulfil personal requirements. The cost of purchasing energy in conventional system for the whole day of load at bus 26 is “US$. 21.4367”. Cost of purchasing energy for the whole day with transactive approach is “US$. 9.7121” and cost of selling as a prosumer is “US$. 29.9695”. So, the total benefit of prosumer 11 is “US$. 20.2574”.

### 4.2 Case 1 evaluation at 10% TE and without TE across 5 years planning horizon

The energy cost in conventional distribution system with 0% TE for base year is illustrated in Table 4. Table 4 serves as a reference regarding load and purchase cost of energy per day. It is observed that the prosumer load has direct impact on the purchase cost and has phenomenal impact when considering coordinated distribution system planning considering transactive DSO based market. It can be observed that the purchase cost of electricity varies with the load at the respective buses, where DGs will be later installed and thus becomes prosumer. The energy cost in conventional distribution system with 10% TE is illustrated in Table 5, where the cost is substantially reduced with the introduction of DGs. The details regarding Impact of 10% TE on all prosumer buses have illustrated in tables (tableA2 till table A12) in the appendix section.

**Table 5:** Energy Cost in Distribution system with 10% TE for base year.

Prosumer #	kW load per day	kW production per day	kW for scheduling per day	Energy sale per day US$/kWh	Cost of purchase per day US$/kWh	Cost Benefit of day US$/kWh	Total Purchase Cost US$/kWh
“Prosumer 1” at bus 29	359.32	343.64	115.2591	7.491842	15.7070	0	8.21516
“Prosumer 2” at bus 35	82.92	181.55	122.727	6.259072	3.0821	3.176972	0
“Prosumer 3” at bus 39	331.68	800	544.8266	26.16041	9.7405	16.41991	0
“Prosumer 4” at bus 41	16.58	480	461.5795	31.38739	0.4870	30.90039	0
“Prosumer 5” at bus 54	345.048	170.338	22.1229	1.548601	23.6440	0	22.0954
“Prosumer 6 at bus 62	418.24	230.722	50.61256	2.277564	28.3850	0	26.1074
“Prosumer 7” at bus 65	771.13	900	345.6212	21.42852	26.4264	0	4.99788
“Prosumer 8” at bus 14	102.72	142.69	94.1515	4.848802	6.8320	0	1.9832
“Prosumer 9” at bus 52	46.224	113.2	89.02704	6.142863	2.9816	3.161263	0
“Prosumer 10” at bus 22	64.2	113.2	80.4856	6.036421	4.2232	1.813221	0
“Prosumer 11” at bus 26	179.76	495	394.3348	29.96945	9.7121	20.25735	0

### 4.3 Cases (1–3) evaluation at 10%, 15% and 20% TE across 5 years planning horizon

#### 4.3.1 Overall power system scheduling: comparison of 10%–20% TE across cases-1-3

**Fig. 20:**
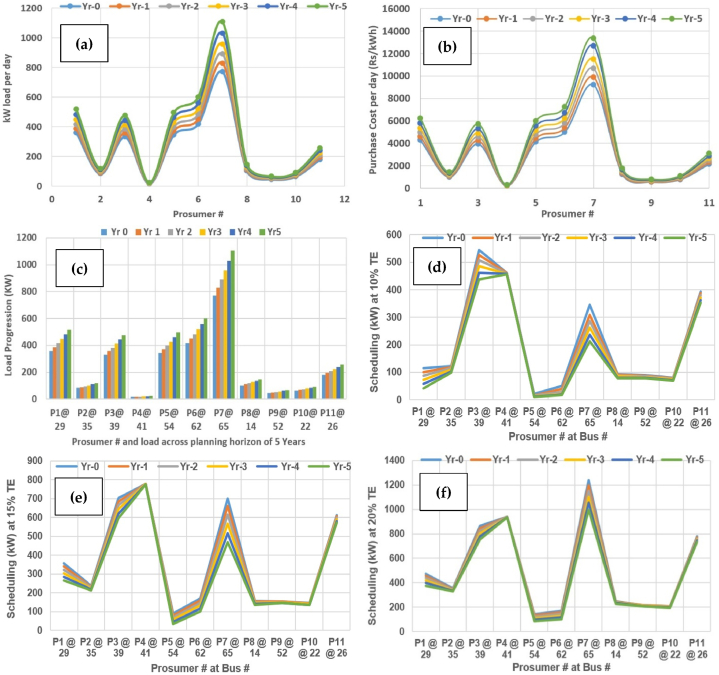
Evaluation across planning horizon of 5 years (a) kW load per day; (b) Purchase cost per day (¢/kWh); (c) Load progression; (d) Scheduling of prosumers at 10% TE; (e) 15% TE; (f) 20% TE.

To keep the discussion relevant and to the point, the further analysis is carried with extrapolation across further 15% and 20% TE across planning horizon expanded around 5 years. The evaluation of various parameters has evaluated across planning horizon of 5 years, as shown in Fig. 20.

It is shown in Fig. 20(a), the kW load progression across planning indicates the increasing load demand. The resulting purchase cost per day (in US'/kWh) also increases by the same manner as shown in Fig. 20(b). Likewise, the load progression across the prosumer buses is illustrated across planning horizon as shown in Fig. 20(c). The scheduling of prosumers in cases 1 at 10% TE, is shown in Fig. 20(d). It can-be observed that there is substantial improvement in scheduling of electric power in KW. The scheduling of prosumers in cases 2 at 15% TE, is shown in Fig. 20(e), where the scheduling impact is more prominent in favor of energy management as compare to case-1 at 10% TE. The scheduling of prosumers in cases 3 at 20% TE, is shown in Fig. 20(f), where the scheduling is much better than the two cases in case-3. It can be observed that the consumer benefit increases as the percentage of TE increases from 10% to 15% and to 20% across each year separately for comparison purposes, respectively. It is also observed that more units are properly scheduled as the % of TE across these cases varies from 10% till 20%, respectively.

The details in-terms of numerical comparison regarding evaluation across planning horizon of 5 years subjected to scheduling of active power (kW) in case-1 at 10% TE is illustrated in Table 6. It is observed that the TE has phenomenal effect on the power in kW across planning horizon and is a suitable alternative to deferral and grid reinforcements. It can-be considered as a viable option for coordinated distribution system planning considering transactive distribution system operator (DSO) based market. Moreover, it is technically and economically viable option for the future distribution mechanisms. Similarly, the evaluation of scheduling of active power (kW) in case-2 at 15% TE is shown in Table 7 and case-3 at 20% in Table 8 replicates the trend of Table 6, respectively.

**Table 6:** Evaluation across planning horizon of 5 years: Scheduling (kW) across 10% TE.

Prosumer#/Yr.	Yr-0; 10% TE	Yr-1; 10% TE	Yr-2; 10% TE	Yr-3; 10% TE	Yr-4; 10% TE	Yr-5; 10% TE
P1 at bus 29	115.2591	100.3222	87.30304	73.48151	58.3816	42.65835
P2 at bus 35	122.727	119.259	114.9338	110.3973	103.2597	99.08133
P3 at bus 39	544.8266	526.9743	507.2239	485.1485	462.2186	437.4524
P4 at bus 41	461.5795	461.1343	460.1785	458.5549	457.3335	455.9769
P5 at bus 54	22.1229	16.5961	15.45165	14.15206	12.20625	11.26361
P6 at bus 62	50.61256	40.3195	28.4028	21.8222	19.59307	18.33014
P7 at bus 65	345.6212	307.8747	285.9411	263.1341	237.6051	212.4753
P8 at bus 14	94.1515	91.6068	88.52951	85.20835	81.17964	77.66897
P9 at bus 52	89.02704	87.944	86.25465	82.64548	80.46079	78.93575
P10 at bus 22	80.4856	78.96	76.97023	74.82041	72.04714	69.90242
P11 at bus 26	394.3348	387.6023	379.7306	371.1475	362.4841	352.2243

**Table 7:** Evaluation across planning horizon of 5 years: Scheduling (kW) across 15% TE.

Prosumer#/Yr.	Yr-0; 15% TE	Yr-1; 15% TE	Yr-2; 15% TE	Yr-3; 15% TE	Yr-4; 15% TE	Yr-5; 15% TE
P1 at bus 29	355.85	339.69477	322.27974	302.92026	283.97828	264.82148
P2 at bus 35	235.06625	231.60602	227.40478	223.34437	217.90386	213.32313
P3 at bus 39	704.0561	685.9797	667.333	644.81	621.7787	597.0842
P4 at bus 41	779.6254	779.0978	779.3204	777.0931	775.9784	774.6858
P5 at bus 54	89.23517	79.81177	69.15388	56.6142	44.17965	34.07529
P6 at bus 62	169.71326	158.17026	145.24173	131.79446	116.16621	100.79584
P7 at bus 65	699.9666	659.6433	615.66773	567.88499	516.99522	467.92341
P8 at bus 14	155.11495	152.42653	149.03329	145.82053	141.21695	136.12577
P9 at bus 52	153.96	152.93393	149.34271	148.07232	145.55624	144.27367
P10 at bus 22	144.78846	143.25309	141.0958	139.20865	136.02669	134.0319
P11 at bus 26	612.9524	606.2222	599.3052	590.2218	581.207	571.3279

**Table 8:** Evaluation across planning horizon of 5 years: Scheduling (kW) across 20% TE.

Prosumer#/Yr.	Yr-0; 20% TE	Yr-1; 20% TE	Yr-2; 20% TE	Yr-3; 20% TE	Yr-4; 20% TE	Yr-5; 20% TE
P1 at bus 29	473.206	456.6777	438.942	419.723	399.302	375.727
P2 at bus 35	352.992	347.1159	342.986	338.373	333.657	328.185
P3 at bus 39	864.162	845.7606	826.041	803.955	781.236	756.379
P4 at bus 41	939.741	938.9165	938.083	936.328	935.435	933.99
P5 at bus 54	142.863	132.9962	122.425	110.847	98.681	85.1594
P6 at bus 62	170.376	158.4167	145.599	131.602	116.84	100.53
P7 at bus 65	1238.32	1196.754	1152.53	1104.45	1053.37	998.689
P8 at bus 14	248.749	245.6738	242.187	238.255	234.283	229.599
P9 at bus 52	215.79	214.4634	212.858	210.938	209.136	206.782
P10 at bus 22	208.398	206.5182	204.318	201.76	199.27	196.178
P11 at bus 26	777.787	770.8192	763.119	754.68	745.576	735.432

#### 4.3.2 Unit price and total production cost: comparison of 10%–20% TE across cases-1-3

**Fig. 21:**
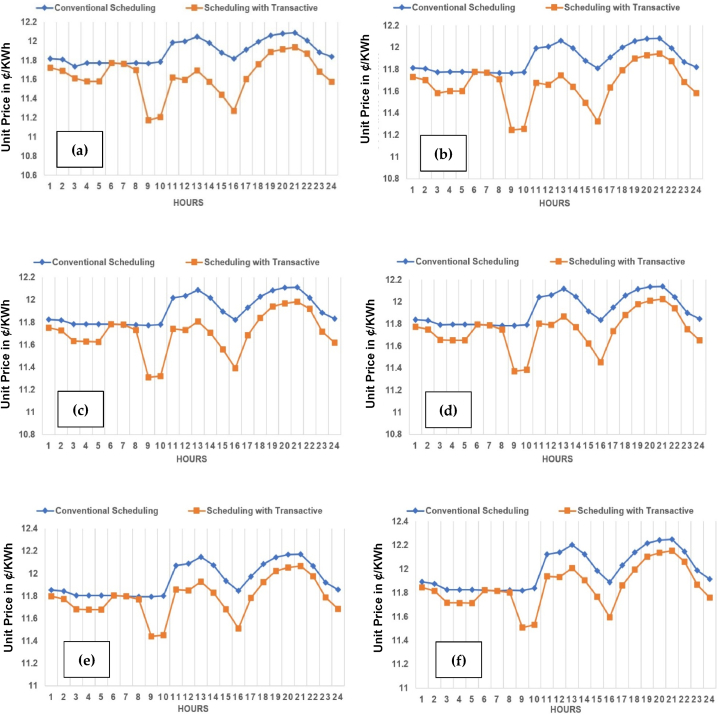
Unit (kWh) cost (in US cents i.e. ¢/kWh) across planning horizon of 5 years (a) Year-0 at 10% TE; (b) Year-1 at 10% TE; (c) Year-2 at 10% TE; (d) Year-3 at 10% TE; (e) Year-4 at 10% TE; (f) Year-5 at 10% TE.

The unit (kWh) cost in ¢/kWh across planning horizon of base to 5 years across three cases of TE at 10%, 15% and 20%, have been evaluated in this subsection. The case-1 of 10% TE is shown across Fig. 21(a)-(f). It can-be observed that there is a phenomenal reduction achieved in unit (kWh) cost in ¢/KWh across each year in particular and overall price as a whole during planning horizon of 5 years. The trend is prominent across all Fig. 21(a)–(f) throughout from base year to 5th year.

**Fig. 22:**
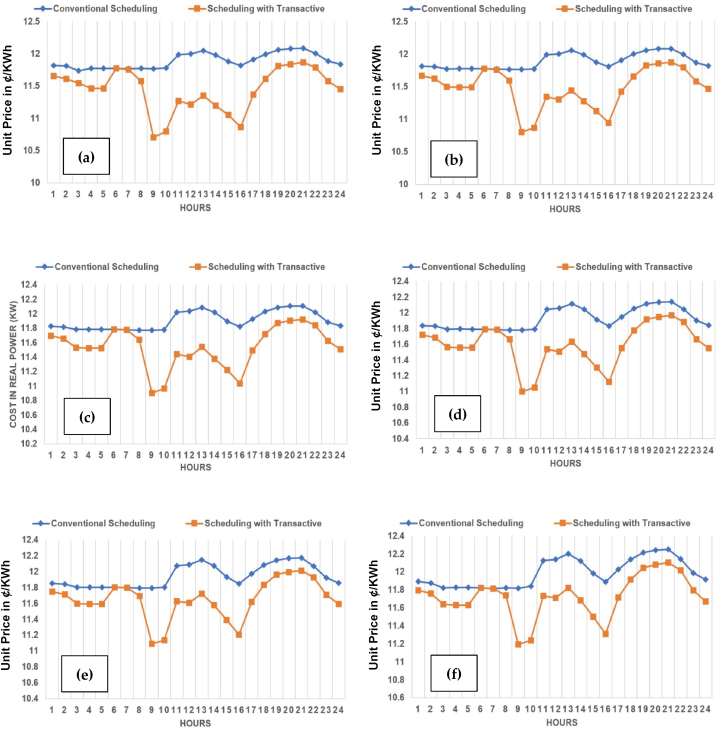
Unit (kWh) cost (¢/kWh) across planning horizon of 5 years (a) Year-0 at 15% TE; (b) Year-1 at 15% TE; (c) Year-2 at 15% TE; (d) Year-3 at 15% TE; (e) Year-4 at 15% TE; (f) Year-5 at 15% TE.

**Fig. 23:**
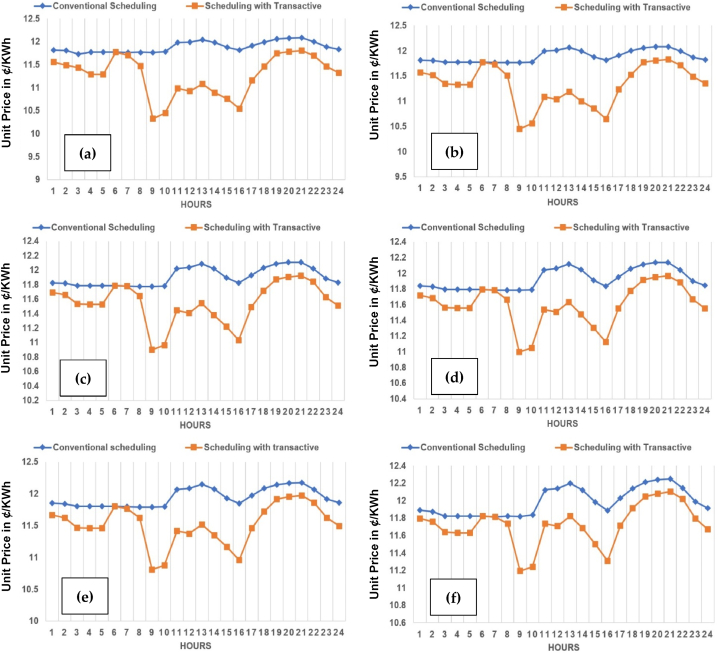
Unit (kWh) cost (¢/kWh) across planning horizon of 5 years (a) Year-0 at 20% TE; (b) Year-1 at 20% TE; (c) Year-2 at 20% TE; (d) Year-3 at 20% TE; (e) Year-4 at 20% TE; (f) Year-5 at 20% TE.

The case-2 of 15% TE is shown across Fig. 22(a)-(f). The case-3 of 20% TE is shown across Fig. 23(a)-(f). The same trend is replicated across each case in favor of both utility and consumers in particular. It is observed, in comparison to the conventional scheduling, the unit price with TE across all three cases have reduced, contributing to prosumer's comfort. Kindly refer to Table-1, Table-2, Table-3 with prosumers classification with 10%, 15% and 20% TE, respectively. Moreover, a reduction of 4–5% is observed in unit price across the maximum (20% TE) penetration in case-3.

Similarly, reduction in total production cost (TPC) across planning horizon of 5 years at 10% TE is illustrated in Table 9. Whereas, reduction in total production cost (TPC) across planning horizon of 5 years at 15% TE and 20% TE, is illustrated in Table 10 and Table 11, respectively. The unit (kWh) cost (¢/kWh) of prosumers in cases 1 at 10% TE, have illustrated in Table 9 shows that the total production cost at prosumers 2, 3, 4, 9, 10 and 11 at respective buses of 35, 39, 41, 52, 22 and 26, is reduced to zero, respectively. This trend indicates that TE has phenomenal effect on the unit price and total production cost across planning horizon.

**Table 9:** Reduction in TPC (US$) across planning horizon of 5 years at 10% TE (Case-1).

Prosumer#/Yr.	Yr-0; 10% TE	Yr-1; 10% TE	Yr-2; 10% TE	Yr-3; 10% TE	Yr-4; 10% TE	Yr-5; 10% TE
P1 at bus 29	8.21516	10.69906	13.38449	16.4706	19.8296	23.36739
P2-4 at bus 35, 39 and 41	0	0	0	0	0	0
P5 at bus 54	22.0954	24.38957	27.9289	31.3652	35.251963	39.242947
P6 at bus 62	26.1074	28.97542	32.463375	36.3032	40.616514	45.384344
P7 at bus 65	4.99788	9.82341	15.80334	22.507	29.80969	37.48122
P8 at bus 14	1.9832	2.652053	3.407528	4.24512	5.197751	6.152751
P9, 11 at bus 52, 26	0	0	0	0	0	0
P10 at bus 22	0	0	0	0	0.361894	0.971122

**Table 10:** Reduction in TPC (US$) across planning horizon of 5 years at 15% TE (Case-2).

Prosumer#/Yr.	Yr-0; 15% TE	Yr-1; 15% TE	Yr-2; 15% TE	Yr-3; 15% TE	Yr-4; 15% TE	Yr-5; 15% TE
P1 at bus 29	0	0	0	0	1.95	5.38191
P2-4 at bus 35, 39 and 41	0	0	0	0	0	0
P5 at bus 54	16.680315	17.3399	22.0932	25.244	25.244	32.3909
P6 at bus 62	19.6573	20.1767	25.6127	28.9701	28.9701	36.6479
P7 at bus 65	0	0	0	0	3.0499	9.11125
P8 at bus 14	0	0	0.20957	1.01784	1.94772	2.98791
P9-11 at bus 52, 22, 26	0	0	0	0	0	0

**Table 11:** Reduction in TPC (US$) across planning horizon of 5 years at 20% TE (Case-3).

Prosumer#/Yr.	Yr-0; 20% TE	Yr-1; 20% TE	Yr-2; 20% TE	Yr-3; 20% TE	Yr-4; 20% TE	Yr-5; 20% TE
P1-4 at bus 29, 35, 39,41	0	0	0	0	0	0
P5 at bus 54	12.314	14.899	17.74	20.818	24.115	27.792
P6 at bus 62	18.472	21.303	24.422	27.794	31.418	33.5456
P7-11 at bus 65,14,52,22,26	0	0	0	0	0	0

Similarly, the unit (kWh) cost (¢/kWh) of prosumers in case-2 at 15% TE results in total production cost at prosumers 1, 2, 3, 4, 7, 8, 9, 10 and 11 at respective buses of 29, 35, 39, 41, 65, 14, 52, 22 and 26, is reduced to zero, respectively. It is observed that TPC in year zero reduces to 42.68 % for 15% TE (case-2) in comparison with case-1 at 10% TE. Whereas, across 5th year, it is 43.30% reduction in TPC is retained for case-2 as compared to case-1, respectively.

Finally, the unit (kWh) cost (¢/kWh) of prosumers in case-3 at 20% TE concludes in total production cost at all prosumers, reduce to ero except prosumers 5 and 6 at respective buses 54 and 62, respectively. It is observed that TPC in year zero reduces to 51.44 % for 20% TE (case-3) in comparison with case-1 at 10% TE. Whereas, across 5th year, it is 59.8% reduction in TPC is retained for case-3 as compared to case-1, respectively. The same trend is replicated in Table 9, Table 10, Table 11 with difference of scale and indicates that the TE has phenomenal effect on the unit price and total production cost across planning horizon.

Thus, TE is a viable economic option from the perspective of deferral and grid reinforcements, while addressing changing load levels, which corresponds to realistic planning problem. The reduction in unit price leads to further reduction in cost of production and will directly benefit both prosumers and utility alike in-terms of low bills and reduction of stress on the grid amid load growth of 5 years horizon, as illustrated in Fig. 23 (a)–(d), respectively.

**Fig. 24:**
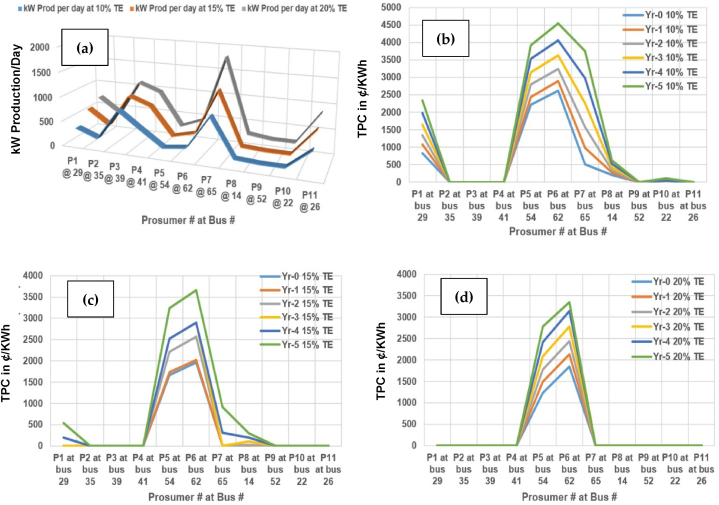
Reduction in TPC across planning horizon. (a) kW production/day; (b) TPC at 10%; (c) 15%; (d) 20%.

It can-be observed that graphically kW production per day increases as the TE by percentage increases from 10% to 20% as shown in Fig. 24(a). For case-1 in Fig. 24(b), the unit (kWh) cost (¢//kWh) of various prosumers decreases to zero. Similarly, in case-2, the impact is more prominent across 15% TE as shown in Fig. 24(c). Finally, unit (kWh) cost (¢/kWh) of many prosumers decreases to zero except a few at a higher scale in case-3 of 20% TE impact as shown in Fig. 24(d).

#### 4.3.3 Total system active power losses: comparison of 10%–20% TE across cases-1-3

The three cases (case 1-3) of TE encapsulating across 10%, 15% and 20%, further adds to reduction of overall active power losses of test distribution system besides efficient power scheduling and TPC. It is observed that in the absence of TE, the overall active power losses are high and with increasing percentage of TE, they are comprehensively reduced. It can be observed in Fig. 24 (a)–(e) that the overall losses are also reduced across the planning horizon and results in achieving overall lower active power losses in comparison with base case (0% TE).

**Fig. 25:**
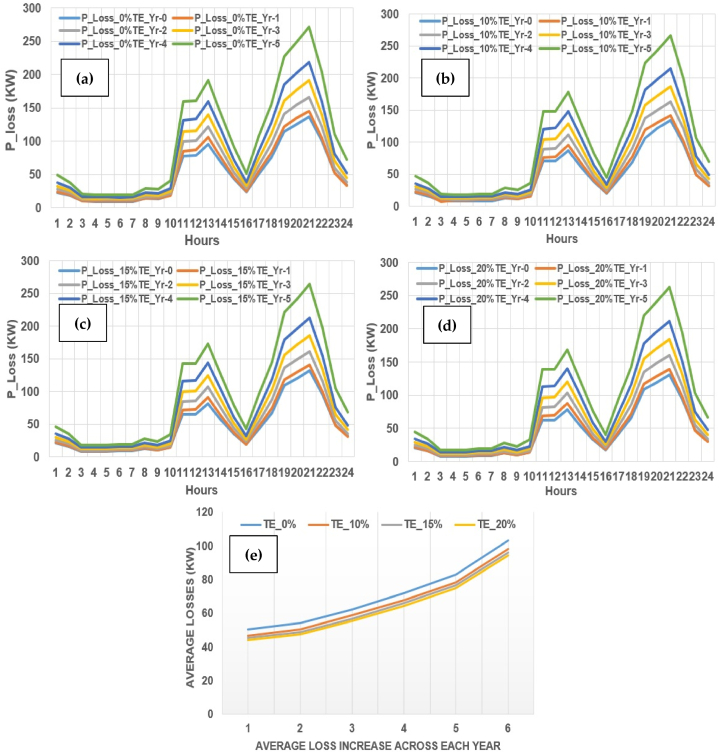
Impact on active power losses across planning horizon.

The base case is shown in Fig. 25(a) with 0% TE across initial year up to the 5th year of the planning horizon of load growth, where the average losses varies from 50.305 KW, 53.863 KW, 62.254 KW, 71.78 KW, 82.613 KW and 102.953 KW, respectively. The case-1 is shown in Fig. 25(b) with 10% TE across initial year up to the 5th year of the planning horizon of load growth, where the average losses varies from 46.593 KW, 50.168 KW, 58.519 KW, 67.617 KW, 78.176 KW and 98.058 KW, respectively. The findings or case-1 is better on numerical basis as compare to the base case.

The case-2 is shown in Fig. 25(c) with 15% TE across initial year up to the 5th year of the planning horizon of load growth, where the average losses varies from 45.247 KW, 48.640 KW, 56.639 KW, 65.835 KW, 76.325 KW and 95.894 KW, respectively. The findings or case-2 is better on numerical basis as compare to the case-1 and base case. The case-3 is shown in Fig. 25(d) with 20% TE across initial year up to the 5th year of the planning horizon of load growth, where the average losses varies from 43.967 KW, 47.297 KW, 55.195 KW, 64.268 KW, 74.647 KW, 94.008 KW, respectively.

The findings or case-3 is better on numerical basis as compare to all compared cases. Finally, a comparative illustration of system losses across all cases have illustrated across planning horizon as shown in Fig. 25(e). The reduction in active power losses leads to further reduction in cost of production and will directly benefit both prosumers and utility alike in-terms of low bills and further reduction of stress on the grid amid load growth across multiple years. It is observed that 12.6% loss reduction is achieved in case-3 (20% TE) in comparison with the base case and is retained to 8.69% across the load growth scenario till the 5th year.

Simulation results show that by the introduction of transactive energy in the conventional distribution system, with the cost benefits for both consumer and utility, system with the transactive energy has shown improved voltage profile and low load losses as compare to the conventional system. With the increase of prosumers participation in the electrical market as observed in the developed system where transactive energy, the customer benefit has increased, the cost to deliver same amount of energy to the same customers has decreased and excess energy from the users has been used thus the efficient use of energy has been seen by involving all available energy resources.

Three transactive penetration levels show greater the penetration greater the benefit of both customer and utility. The system with the lowest transactive level has 10 % DERs connected in the system that has possibility to participate in the scheduling is better than the system without any DERs participating in the system. While the system with 10 % transactive is better than without transactive, 15 % transactive is seen to be better than 10 % and same as 20 % transactive participation is better than 15 % transactive approach applied. It is shown in this research that transactive energy has proven better than the conventional system. maximum 20 % transactive energy participation has been simulated in the research. It is observed that the TE has phenomenal effect on the power scheduling, reduction in total production cost and decrease in active power losses across planning horizon and is a suitable alternative to deferral and grid reinforcements. In future, reliability and reputation index-based market framework will be proposed in future studies.

## 5 Conclusions

In this paper, three transactive penetration levels (10%, 15% and 20%) shows more benefits for both customer and utility in comparison with base case without TE. The system evaluated with three TE cases incorporating DERs connected in the system, resulting better possibility to participate in the scheduling of resources. It is observed that the TE has phenomenal effect on the power in kW across planning horizon and is a suitable alternative to deferral and grid reinforcements. The scheduling of prosumers in various cases shows substantial improvement in scheduling of electric power in favor of energy management. the TE has phenomenal effect on the power in kW across planning horizon and is a suitable alternative to deferral and grid reinforcements. It can-be considered as a viable option for coordinated distribution system planning considering transactive distribution system operator-based market. Moreover, it is technically and economically viable option for the future distribution mechanisms. It can-be observed that there is a phenomenal reduction achieved in unit (kWh) cost in ¢/kWh across each year in particular and overall price as a whole during planning horizon of 5 years. The trend is prominent across base year to 5th year. Moreover, with difference of scale, the TE has phenomenal effect on the unit price and total production cost across planning horizon. It is found that 51.44 % reduction in total production cost of energy is achieved with reduction of per unit price around 5%. Whereas 12.6% reduction of losses have achieved across maximum TE penetration case. It is also observed that the three cases of TE encapsulating across 10%, 15% and 20%, further adds to comprehensive reduction of overall active power losses of test distribution system in comparison with the base case. The reduction in active power losses leads to further reduction in cost of production and will directly benefit both prosumers and utility alike in-terms of low bills and further reduction of stress on the grid amid load growth across multiple years. Hence, it is viable option from the perspective of techno-economic evaluation, primarily for the future distribution mechanisms. Transactive system is developed for IEEE 69 bus system that has low system load profile. Future work includes blockchain enabled hybrid solar system with battery, wind energy system, biogas and other renewables energy system participation in the system and their effect on the system in the perspective of voltage profile, losses, energy efficiency and cost benefit, across multiple planning horizons.

## Funding

10.13039/501100000725The authors extend their appreciation to 10.13039/501100000725the deputyship for research & innovation, ministry education in Saudi Arabia for funding this research work through 10.13039/501100000725the project number (ifp-2022-44).

## Data availability

Data will be made available on request.

## Additional information

No additional information is available for this paper.

## CRediT authorship contribution statement

**Mustafa Tariq:** Writing – review & editing, Writing – original draft, Visualization, Validation, Software, Methodology, Investigation, Conceptualization. **Zafar A. Khan:** Writing – review & editing, Supervision, Software, Resources, Methodology, Investigation, Conceptualization. **Syed Ali Abbas Kazmi:** Writing – original draft, Methodology, Investigation, Conceptualization. **Abdullah Altamimi:** Writing – review & editing, Resources, Project administration, Formal analysis, Data curation. **Bader Alharbi:** Writing – review & editing, Supervision, Software, Resources, Methodology, Investigation, Formal analysis, Data curation, Conceptualization. **Hamoud Alafnan:** Writing – review & editing, Supervision, Software, Resources, Methodology, Investigation, Conceptualization. **Halemah Alshehry:** Writing – review & editing, Supervision, Software, Resources, Methodology, Investigation, Formal analysis, Data curation, Conceptualization.

## Declaration of competing interest

The authors declare that they have no known competing financial interests or personal relationships that could have appeared to influence the work reported in this paper.

## Figures and Tables

**Table A1:** Features of the proposed TE framework with respect to literature review.

Ref	TE Energy Management Framework	Load Variations and Prosumer consideration	Tariff and cost to benefit analysis	DSO and System Losses Consideration	kWh cost & Market impact	Flexibility of Cases by %	Load Peak shaving	MG Consideration and REG	Loop/Mesh Configuration / Deferral
[3]	Scheduling	✓	**✓**	**✓**	**✓**		✗	**✓**	✗
[4]	Scheduling	✓	**✓**	**✓**	**✓**	✗	✗	**✓**	✗
[5]	Scheduling	**✓**	**✓**	**✓**	**✓**	✗	✗	**✓**	✗
[6]	Scheduling	**✓**	**✓**	**✓**	**✓**	**✓**	✗	✗	✗
[7]	Scheduling	**✓**	**✓**	**✓**	**✓**	✗	✗	**✓**	✗
[8]	Scheduling	**✓**	**✓**	**✓**	**✓**	✗	✗	✗	✗
[10]	Scheduling	**✓**	**✓**	**✓**	**✓**	**✓**	**✓**	✗	✗
[11]	Scheduling	**✓**	**✓**	**✓**	**✓**	✗	✗	**✓**	✗
[16]	Scheduling	**✓**	**✓**	**✓**	**✓**	✗	✗	✗	✗
[18]	Scheduling	**✓**	**✓**	**✓**	**✓**	✗	✗	**✓**	✗
[21]	Scheduling	**✓**	**✓**	**✓**	**✓**	✗	✗	**✓**	✗
[22]	Scheduling	**✓**	**✓**	**✓**	**✓**	✗	✗	✗	✗
[25]	Scheduling	**✓**	**✓**	**✓**	**✓**	✗	✗	✗	✗
[26]	Planning	✗**/✓**	**✓**	**✓**	**✓**	✗	✗	**✓**	✗
[P]	Scheduling and Planning	**✓**	**✓**	**✓**	**✓**	**✓**	**✓**	**✓**	**✓**

**Table A2:** Impact of 10% TE on prosumer-1 @ bus 29 across 5-year planning horizon.

Prosumer-1 @ Bus 29 (Parameters)	Year-1	Year-2	Year-3	Year-4	Year-5
Electricity cost without TE (US$)	6867.65	7203.819	7757.696	8354.784	9020.816
Electricity cost with TE (US$)	6525.05	6867.647	7415.094	8006.109	8664.927
Electricity cost Difference (US$)	342.593	336.172	342.602	348.675	355.889
Energy needs without TE (kWh)	386.85	416.54	448.57	483.122	520.40
Energy needs with TE (kWh)	343.64	343.64	343.64	343.64	343.64
Energy needs met with PV (kWh)	100.32	87.303	73.48	58.38	42.658
Energy purchase without TE (US$)	46.0206	46.92699	53.5248	57.7394	62.1940
Energy purchase with TE (US$)	17.22	19.0592	21.2469	23.6244	26.1401
Overall Difference: (US$)	28.8006	27.86779	32.2779	34.115	36.0539
Cost of selling from consumer (US$)	6.52093	5.6747	4.7763	3.7948	2.77279
Cost difference in purchase of energy (US$):	28.8006 +6.52093 = 35.3216	27.86779 +5.6747 = 33.54249	32.2779 +4.7763 = 37.0542	34.115 +3.7948 = 37.9098	36.0539 +2.77279 = 38.8266
Impact on total consumer's expense (US$):	46.0206 −35.3216 = 10.6991	46.92699 −33.54249 = 13.38.45	53.5248 −37.0542 = 16.4706	57.7394 −37.9098 = 19.8296	62.1940 −38.8266 = 23.36739

**Table A3:** Impact of 10% TE on prosumer-2 @ bus 35 across 5-year planning horizon.

Prosumer-2 @ Bus 35 (Parameters)	Year-1	Year-2	Year-3	Year-4	Year-5
Energy needs without TE (kWh)	89.139	95.824	103.01	110.73	119.042
Energy needs with TE (kWh)	181.55	181.55	181.55	181.55	181.55
Energy needs met with PV (kWh)	119.22	114.93	110.39	103.25	99.08
Energy purchase without TE (US$)	10.6041	11.417	12.2920	13.2325	14.2279
Energy purchase with TE (US$)	3.36736	3.682	4.0196	4.3841	4.7651
Overall Difference: (US$)	7.23674	7.735	8.2724	8.8484	9.4628
Cost of selling from consumer (US$):	2.71314	2.17981	1.61066	0.8821	0.288
Cost difference in purchase of energy (US$):	7.23674 +2.71314 = 9.95	7.735 +2.17981 = 9.915	8.2724 +1.61066 = 9.883	8.8484 +0.8821 = 9.7305	9.4628 +0.288 = 9.7508
Impact on total consumer's expense (US$):	10.6041 −9.95 = 0.6541	11.41665 −9.915 = 1.5017	12.292 −9.883 = 2.409	13.2325 −9.7305 = 3.502	14.2279 −9.7508 = 4.4771

**Table A4:** Impact of 10% TE on prosumer-3 @ bus 39 across 5-year planning horizon.

Prosumer-3 @ Bus 39 (Parameters)	Year-1	Year-2	Year-3	Year-4	Year-5
Energy needs without TE (kWh)	356.556	383.756	412.045	442.948	476.17
Energy needs with TE (kWh)	800	800	800	800	800
Energy needs met with DG (kWh)	526.97	506.7	485.554	462.21	437.45
Energy purchase without TE (US$)	42.417	45.667	49.1680	52.94114	56.9117
Energy purchase with TE (US$)	10.476	11.282	12.1462	13.0779	14.0587
Overall Difference (US$):	31.941	34.385	37.0218	39.86324	42.853
Cost of selling from consumer (US$):	14.81872	13.4825	11.1409	9.1085	6.939
Cost difference in purchase of energy (US$):	31.941 +14.81872 = 46.75972	34.385 +13.485 = 47.87	37.0218 +11.1409 = 48.1627	39.86324 +9.1085 = 48.97174	42.853 +6.939 = 49.792
Impact on total consumer's expense (US$):	42.41 −46.75972 = -4.34272	45.667 −47.87 = -2.203	49.1680 −48.1627 = 1.0053	52.94114 −48.97175 = 3.9694	56.9117 −49.792 = 7.1197

**Table A5:** Impact of 10% TE on prosumer-4 @ bus 41 across 5-year planning horizon.

Prosumer-4 @ Bus 41 (Parameters)	Year-1	Year-2	Year-3	Year-4	Year-5
Energy needs without TE (kWh)	17.82	19.165	20.60	22.14	23.808
Energy needs with TE (kWh)	480	480	480	480	480
Energy needs met with DG (kWh)	461.13	460.178	458.55	457.33	455.97
Energy purchase without TE (US$)	2.1208	2.2833	2.4584	2.647	2.8455
Energy purchase with TE (US$)	0.5238	0.5641	0.6073	0.6539	0.7029
Overall Difference: (US$)	31.3571	31.29214	31.18172	31.09.86	31.0064
Cost of selling from consumer (US$):	30.8333	30.72804	30.57442	30.4447	30.3035
Cost difference in purchase of energy (US$):	31.3571 −30.8333 = 0.5238	31.29214 −30.72804 = 0.5641	31.18172 −30.57442 = 0.6073	31.0986 −30.4447 = 0.6539	31.0064 −30.30.35 = 0.7029
Impact on total consumer's expense (US$):	2.1208 −0.5238 = 1.597	2.28.33 −0.5641 = 1.7191	2.45.84 −0.6073 = 1.8511	2.64.7 −0.6539 = 1.9931	2.84.55 −0.7029 = 2.1426

**Table A6:** Impact of 10% TE on prosumer-5 @ bus 54 across 5-year planning horizon.

Prosumer-5 @ Bus 54 (Parameters)	Year-1	Year-2	Year-3	Year-4	Year-5
Energy needs without TE (kWh)	370.926	398.746	428.65	460.80	495.36
Energy needs with TE (kWh)	170.338	170.338	170.338	170.338	170.338
Energy needs met with PV (kWh)	16.59	15.45	14.15	12.20	11.26
Energy purchase without TE (US$)	44.3075	47.7149	51.3873	55.34638	59.4973
Energy purchase with TE (US$)	24.3896	27.9289	31.36526	35.252	39.243
Overall Difference (US$):	19.9179	19.786	20.02204	20.09204	20.2543
Cost of selling from consumer (US$):	1.1617	1.0816	0.9906	0.8544	0.7884

**Table A7:** Impact of 10% TE on prosumer-6 @ bus 62 across 5-year planning horizon.

Prosumer-6 @ Bus 62 (Parameters)	Year-1	Year-2	Year-3	Year-4	Year-5
Energy needs without TE (kWh)	449.608	483.329	519.57	558.54	600.438
Energy needs with TE (kWh)	230.722	230.722	230.722	230.722	230.722
Energy needs met with PV (kWh)	40.319	28.40	21.822	19.59	18.33
Energy purchase without TE (US$)	53.70609	57.83626	62.28769	67.0865	72.1180
Energy purchase with TE (US$)	30.7898 −1.8143 = 28.9755	33.7415 −1.2781 = 32.4634	37.2852 −0.982 = 36.3032	41.4982 −0.8816 = 40.6166	46.2092 −0.8248 = 45.3844
Overall Difference (US$):	24.73059	33.1056	25.9845	26.4699	26.7256
Cost of selling from consumer (US$):	1.8143	1.2781	0.982	0.8816	0.8248

**Table A8:** Impact of 10% TE on prosumer-7 @ bus 65 across 5-year planning horizon.

Prosumer-7 @ Bus 65 (Parameters)	Year-1	Year-2	Year-3	Year-4	Year-5
Energy needs without TE (kWh)	828.96	891.137	957.972	1029.82	1107.057
Energy needs with TE (kWh)	900	900	900	900	900
Energy needs met with DG (kWh)	307.87	285.94	263.13	237.60	212.47
Energy purchase without TE (US$)	99.0206	106.6356	114.8429	123.69077	132.9675
Energy purchase with TE (US$)	24.73059	33.53179	38.8213	44.54122	50.6547
Overall Difference: (US$)	99.0206–9.84334 = 89.203	90.8321	92.3359	95.46385	95.4862
Cost of selling from consumer (US$):	28.9116–19.0882 = 9.8234	15.80349	22.507	28.22692	37.48122

**Table A9:** Impact of 10% TE on prosumer-8 @ bus 14 across 5-year planning horizon.

Prosumer-8 @ Bus 14 (Parameters)	Year-1	Year-2	Year-3	Year-4	Year-5
Energy needs without TE (kWh)	110.424	118.706	127.609	137.179	147.467
Energy needs with TE (kWh)	142.69	142.69	142.69	142.69	142.69
Energy needs met with PV (kWh)	91.606	88.52	85.2	81.17	77.68
Energy purchase without TE (US$)	13.1659	14.1764	15.26566	16.4398	17.6728
Energy purchase with TE (US$)	7.3698	7.9668	8.6333	9.3785	10.1527
Overall Difference (US$):	13.1659 −2.6521 = 10.5138	14.1764 −3.4076 = 10.7688	15.26566 −4.2451 = 11.02056	16.4398 −5.1977 = 11.2421	17.6728 −6.1528 = 11.52
Cost of selling from consumer (US$):	4.7177	4.5592	4.3882	4.1808	3.9999

**Table A10:** Impact of 10% TE on prosumer-9 @ bus 52 across 5-year planning horizon.

Prosumer-9 @ Bus 52 (Parameters)	Year-1	Year-2	Year-3	Year-4	Year-5
Energy needs without TE (kWh)	49.69	53.41	57.424	61.731	66.36
Energy needs with TE (kWh)	113.20	113.20	113.20	113.20	113.20
Energy needs met with PV (kWh)	87.943	86.25	82.20	80.46	78.935
Energy purchase without TE (US$)	5.9246	6.3794	6.8695	7.3979	7.9527
Energy purchase with TE (US$)	3.22345	3.4917	3.7800	4.0912	4.4141
Overall Difference (US$):	2.70115	2.8877	3.0895	3.3067	3.5386
Cost of selling from consumer (US$):	2.84465	2.4598	1.9225	1.4606	1.0324

**Table A11:** Impact of 10% TE on prosumer-10 @ bus 22 across 5-year planning horizon.

Prosumer-10 @ Bus 22 (Parameters)	Year-1	Year-2	Year-3	Year-4	Year-5
Energy needs without TE (kWh)	69.01	74.19	79.755	85.737	92.16
Energy needs with TE (kWh)	113.20	113.20	113.20	113.20	113.20
Energy needs met with PV (kWh)	78.955	76.97	74.82	72.047	69.90
Energy purchase without TE (US$)	8.2286	8.8603	9.5410	10.2748	11.0455
Energy purchase with TE (US$)	4.5592	4.93225	5.3328	5.76544	6.2138
Overall Difference (US$):	3.6694	3.92805	4.2082	4.50936	4.8317
Cost of selling from consumer (US$):	1.3624	0.84	0.24	0.36194	0.9712

**Table A12:** Impact of 10% TE on prosumer-11 @ bus 26 across 5-year planning horizon.

Prosumer-11 @ Bus 26 (Parameters)	Year-1	Year-2	Year-3	Year-4	Year-5
Energy needs without TE (kWh)	193.242	207.74	223.315	240.06	258.069
Energy needs with TE (kWh)	495	495	495	495	495
Energy needs met with DG (kWh)	387.60	379.73	371.14	362.48	352.22
Energy purchase without TE (US$)	23.04034	24.80885	26.7149	28.7697	30.9274
Energy purchase with TE (US$)	10.4530	11.2729	12.1511	13.1009	12.6856
Overall Difference (US$):	12.58734	13.536	14.5638	15.6688	18.2418
Cost of selling from consumer (US$):	29.4577	28.8616	28.20721	27.5488	26.7690

## Appendix

All the details of evaluations subjected to 10% TE impact have illustrated from table A2 for prosumer #1 until table A12 for prosumer#11, respectively.

## List of abbreviations

CM: contribution metric
CP: consumer points
CMES: Comprehensive energy management system
CPP: Critical peak pricing
CPR: Critical peak rebate tariffs
CVaR: Conditional value at risk
DA: Day ahead
DESS: Distributed energy storage system
DSM: Demand side management
DSO: distribution system operator
DR: Demand response
DERs: distributed energy resources
EPSO: Enhanced Particle Swarm Optimization algorithm
HVAC: Heat ventilation, and air-conditioning approach
GA: Genetic Algorithm
ITD: integrated transmission and distribution
MCS: Monte Carlo Simulation
MDS/MDN: Mesh distribution system/Mesh distribution network
MG: Microgrid
PP: Producer points
RT: Real Time
US ¢: United States Cents
US $: United States Dollar = 100 United States Cents
RTP: Real time pricing tariffs
TE: Transactive energy
ToU: Time-of-use
VPP: Virtual Power Plant
